# Chemosensory response to Pt-based chemotherapeutics *via* bitter taste receptors in vitro reveals a new mechanism for bitter taste disorders

**DOI:** 10.1038/s41598-026-35636-w

**Published:** 2026-01-20

**Authors:** Sofie Zehentner, Agnes Mistlberger-Reiner, Philip Pirkwieser, Noreen Orth, Valerie Boger, Kristin Kahlenberg, Johanna Kreißl, Christoph Grimm, Jakob Peter Ley, Veronika Somoza

**Affiliations:** 1 Institute of Physiological Chemistry, Faculty of Chemistry, University of Vienna, Vienna, Austria; 2 Vienna Doctoral School in Chemistry (DoSChem), University of Vienna, Vienna, Austria; 3 Leibniz Institute for Food Systems Biology at the Technical University of Munich, Freising, Germany; 4 Department of General Gynecology and Gynecologic Oncology, Comprehensive Cancer Center Vienna, Gynecologic Cancer Unit, Medical University of Vienna, Vienna, Austria; 5 Symrise AG, Research Biobased Ingredients, Research & Technology, Food & Beverage, Taste, Nutrition & Health, Holzminden, Germany; 6 Chair of Nutritional Systems Biology, School of Life Science, Technical University of Munich, Freising, Germany

**Keywords:** Pt-based chemotherapeutic drugs, Bitter taste disorders, Bitter taste receptors (TAS2Rs), Homoeriodictyol, Bitter masker, Cellular uptake, Biochemistry, Cancer, Drug discovery, Oncology

## Abstract

Chemotherapeutics like platinum (Pt)-based drugs cause bitter taste disorders, which impair patients’ food intake and quality of life. However, the role of bitter taste receptors (TAS2Rs) remains unexplored, limiting the development of targeted treatments for this clinically impactful side effect. By performing a TAS2R-sensitive bitterness assay based on human gastric parietal cells (HGT-1), we evaluated the bitterness of Pt-based agents and the counteracting potential of the bitter-masking sodium salt of the flavanone homoeriodictyol (Na-HED). Here, we report that carboplatin (50–750 µM) and cisplatin (5–50 µM) elicited dose-dependent cellular bitter responses, with cisplatin evoking a stronger effect. Functional involvement of TAS2R4 and TAS2R5 was confirmed by CRISPR-Cas9 knockout- and siRNA knockdown-experiments. Na-HED reduced the cellular bitter response of 200 µM carboplatin (− 76% ± 11%) and 50 µM cisplatin (– 75% ± 15%). Additionally, this study provides evidence that TAS2Rs modulate the cellular uptake of cytotoxic Pt-based agents, underlining the importance of these chemoreceptors at the cellular level. In conclusion, our results demonstrate a functional role of TAS2Rs in the bitter response induced by Pt-based agents and a counteracting potential of Na-HED, suggesting the development of TAS2R-targeted treatment strategies to address chemotherapy-induced bitter taste hypersensitivity and bitter phantogeusia.

**Supplementary Information:**

The online version contains supplementary material available at 10.1038/s41598-026-35636-w.

## Introduction

Taste disorders affecting the perception of bitter taste are prevalent adverse effects of chemotherapy^1–4^ and are negatively related to energy intake^5^, body weight and quality of life^6^, all of which have a significant impact on cancer treatment outcomes^7–9^. A multifactorial and heterogeneous etiology of taste dysfunctions in cancer patients is hypothesized^10–12^. Yet, the mechanisms underlying bitter taste alterations and bitter phantogeusia, i.e., the perception of bitterness in the absence of external stimuli, remain unclear, especially regarding the role of bitter taste receptors (TAS2Rs). This represents a significant research gap that contributes to the current lack of effective treatment options for bitter taste disorders.

As oncological patients are more likely to report that everything tastes bitter when experiencing a metallic taste sensation^13^, which is not well defined on a molecular level^59^ and often associated with bitter taste^14^, chemotherapeutics containing a metal compound such as platinum (Pt)^15^ were of particular interest in our study. In general, to elicit a bitter taste sensation, compounds need to bind to one or more of the approximately 26 human TAS2Rs^16^, of which 25 are proven to be expressed in taste cells of taste buds on the tongue^17^.

Various structurally diverse agonists trigger the activation of TAS2Rs^18–20^. However, a bitter taste quality of chemotherapeutic drugs, including those based on metals, has not been proven so far, as a sensory evaluation of these intravenously administered, cytotoxic agents is not ethically justifiable. Closing this research gap by in vitro studies could open new avenues for developing effective treatment strategies to address chemotherapy-induced bitter taste hypersensitivity and bitter phantogeusia, ultimately enhancing patients’ food intake and quality of life.

For intravenously given chemotherapeutics, various pathways have been suggested by which these drugs may enter the oral cavity and influence taste sensations, e.g., *via* saliva, crevicular fluid, or blood/exudate due to mucosal damage caused by oral cavity infection^21^. Consequently, chemotherapeutic drugs are postulated, but not yet demonstrated, to exhibit bitter and/or metallic taste^15,21^, and have been reported to modulate *TAS2R5* gene expression, linked to phantogeusia^22^.

Specifically, since Pt is detectable in saliva for several hours following Pt-based chemotherapy^23,24^, we first hypothesize that intravenously administered Pt-based chemotherapeutic drugs, once transported into saliva, elicit a bitter taste sensation *via* activation of TAS2Rs, representing a mechanistic basis for bitter taste hypersensitivity and bitter phantogeusia. Strictly speaking, Pt-based drugs are external stimuli, but we describe the taste phenomenon under investigation as phantogeusia because the drug is thought to reach the oral cavity *via* saliva after intravenous administration. Assuming that a chemosensory response is actually triggered by chemotherapeutic drugs *via* TAS2Rs, we propose that the sodium salt of homoeriodictyol (Na-HED; sodium salt of 3’-methoxy-4’,5,7-trihydroxyflavanone), a bitter-masking flavanone isolated from Herba Santa (*Eriodictyon californicum* (H. & A.) Torr.)^25^, which is known to be an antagonist of TAS2R20, TAS2R31, TAS2R43, and TAS2R50^26^ and an agonist of TAS2R14^26,27^ and TAS2R39^27^ counteracts this chemical sensing. By investigating our second hypothesis that Na-HED reduces the bitter response induced by Pt-based chemotherapeutic agents in vitro, this study aims to provide an experimental basis for the potential future development of Na-HED-based treatments for increased bitter taste sensitivity and bitter phantogeusia. Moreover, TAS2Rs were recently shown to co-regulate the cellular uptake of metal ions^28,29^, rendering TAS2Rs interesting candidates for modulating the uptake of metal-based chemotherapeutics. As our third main hypothesis, we therefore propose that TAS2Rs influence the cellular uptake of carboplatin and cisplatin, potentially *via* chemosensation.

To verify these hypotheses, immortalized human gastric parietal (HGT-1) cells, a validated surrogate model for assessing the bitterness of compounds^26,30–32^, were used to explore (i) whether carboplatin and cisplatin elicit a cellular bitter response in this in vitro model, while Na-HED counteracts the induced cellular bitter response, (ii) whether carboplatin and cisplatin influence the gene expression levels of *TAS2R*s, and (iii) whether specific TAS2Rs play a functional role in this context, which was evaluated by CRISPR-Cas9 knockout (KO) and siRNA knockdown (KD) experiments.

Additionally, (iv) the cellular uptake of carboplatin and cisplatin in absence of specific TAS2Rs as well as without and in the presence of Na-HED was evaluated. Since the intravenously administered amount of the two tested Pt-based chemotherapeutic agents used in cancer therapy differs between cisplatin and carboplatin by a ratio of 1:4^33^, this concentration ratio was considered in order to compare the effect sizes in all in vitro studies.

## Results

### Carboplatin and cisplatin stimulate the bitter response in an in vitro cell model for bitterness assessment

**Fig. 1:**
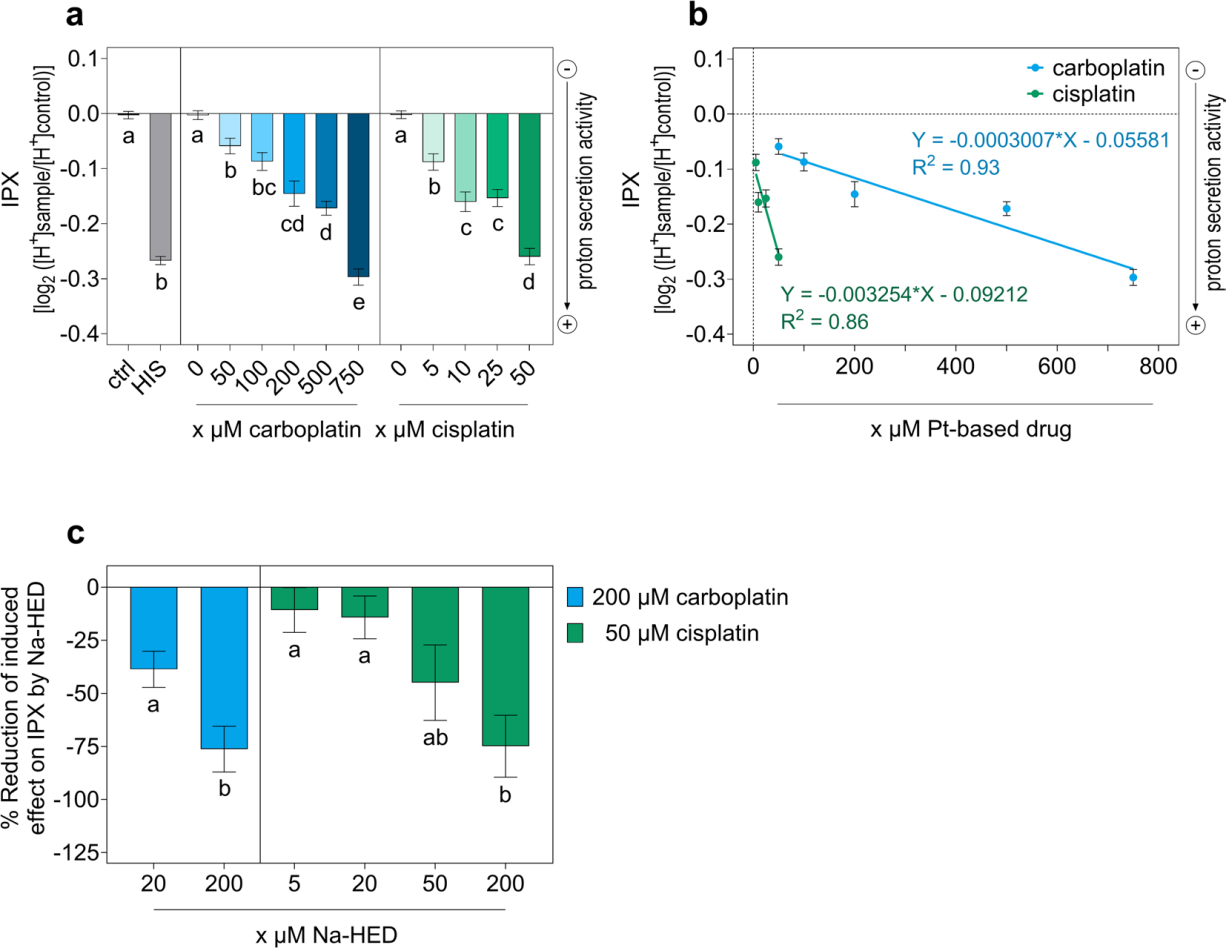
TAS2R-mediated cellular bitter response triggered by Pt-based drugs and reducing effects of Na-HED. **(a)** Intracellular proton index (IPX) of HGT-1 cells wild-type (WT) after incubation with carboplatin or cisplatin in various concentrations for 24 h. 1 mM histamine (HIS) was used as positive control for functional H^+^-release during measurement, with Krebs-Ringer-HEPES buffer (KRHB) alone serving as a control (ctrl). Data are presented as mean ± SEM, *n* = 9–23, tech. repl. = 4–6; Statistics: Welch’s ANOVA with Dunnett’s T3 multiple comparisons test for both treatment groups, and two-tailed Welch’s t-test for ctrl *versus* HIS. Significant differences within each treatment group are indicated by different letters (*p* ≤ 0.05). **(b)** Linear regression of IPX of HGT-1 WT cells after incubation with carboplatin or cisplatin in various concentrations for 24 h. Data are presented as mean ± SEM, *n* = 9–15, tech. repl. = 4–6. **(c)** Relative effect of Na-HED in reducing the carboplatin- or cisplatin-induced effect on IPX in HGT-1 WT cells after co-incubation with Na-HED at various concentrations and either 200 µM carboplatin or 50 µM cisplatin for 24 h. Data are presented as mean ± SEM, *n* = 5, tech. repl. = 4–6. Statistics: two-tailed t-test for co-incubations with Na-HED plus carboplatin, and one-way ANOVA with Holm-Šídák’s multiple comparisons test for co-incubations with Na-HED plus cisplatin. Significant differences within each treatment group are indicated by different letters (*p* ≤ 0.05).

As a substitute for sensory tasting to assess bitter-tasting and bitter-modulating compounds, we used HGT-1 cells as a validated in vitro model of TAS2R-dependent proton secretion^26,31,32^ to evaluate the cellular bitter response induced by carboplatin and cisplatin. In this in vitro model, increased proton secretion activity, indicated by the decrease in intracellular proton index (IPX), is known to be a functional response to the activation of TAS2Rs in HGT-1 cells^26,31,32^. Following a 24-h exposure to carboplatin or cisplatin at concentrations that ensure cell viability (see Supplementary Table S1 online), bitter response of HGT-1 wild-type (WT) cells was evoked in a dose-dependent manner (Fig. 1a).

The dose-dependency showed a linear regression for both compounds (Fig. 1b), while cisplatin appeared to have a stronger dose-related impact than carboplatin. Considering the 1:4 concentration ratio between the two Pt-based chemotherapeutics administered in cancer therapy^33^, the maximal cisplatin effect observed at 50 µM (IPX − 0.26 ± 0.01) showed a stronger IPX reduction by about 79% ± 10% (two-tailed Mann-Whitney test; *p* ≤ 0.0001) compared to 200 µM carboplatin (IPX − 0.15 ± 0.02). This emphasizes the greater impact of cisplatin in inducing a bitter response in the cellular model.

### The sodium salt of HED, a bitter-masking flavanone, counteracts the cellular bitter response triggered by carboplatin or cisplatin

Next, the potential of the bitter-masking compound Na-HED to counteract the cellular bitter response induced by Pt-based drugs in HGT-1 WT cells was investigated. The bitter-masking compound Na-HED counteracted the carboplatin- and cisplatin-induced effects on IPX in a dose-dependent manner (Fig. 1c). Co-incubation of HGT-1 WT cells with 200 µM Na-HED reduced the cellular bitter response caused by 200 µM carboplatin (76% ± 11%) or 50 µM cisplatin (75% ± 15%) at a similar relative level (two-tailed t-test, *p* = 0.94). Na-HED per se did not affect the IPX after 24-h incubation (see Supplementary Fig. S1a online) at any of the concentrations tested (5, 20, 50, 200 µM). These results demonstrate that Na-HED, an antagonist of TAS2R20, 31, 43 and 50^26^, reduces the cellular bitter response induced by carboplatin and cisplatin.

### Carboplatin and cisplatin influence the gene expression of *TAS2R*s in HGT-1 cells

**Fig. 2:**
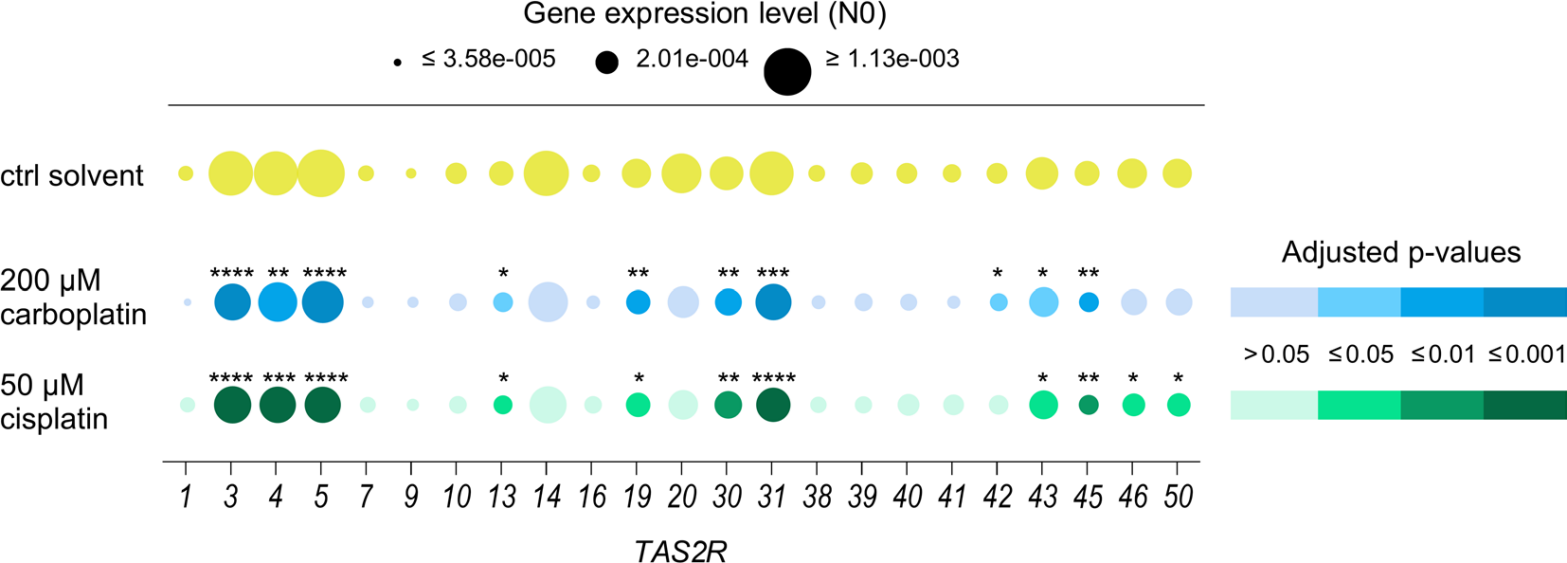
Effects of Pt-based drugs on gene expression of *TAS2R*s. The bubble blot shows gene expression levels of the 23 *TAS2R*s analyzed in HGT-1 wild-type (WT) cells after treatment with solvent control (ctrl solvent), 200 µM carboplatin, or 50 µM cisplatin for 18 h. All gene expression levels (N0) were normalized to three housekeeping genes (*GAPDH*, *PPIA*, *TBP*). The color shades of 200 µM carboplatin and 50 µM cisplatin show the adjusted p-values obtained by statistical analysis of gene expression levels (N0) of HGT-1 WT cells after treatment with 200 µM carboplatin or 50 µM cisplatin for 18 h compared to expression levels in solvent-treated control HGT-1 WT cells (ctrl solvent) (*n* = 3, tech. repl. = 2–3; except *TAS2R9*: only measurable in 1/3 carboplatin-treated and 2/3 cisplatin-treated biological replicates; *TAS2R1* and *TAS2R40*: after cisplatin treatment, detectable in two biological replicates and in the third only in 1/3 tech. repl.; *TAS2R38*: after carboplatin treatment, detectable in two biological replicates and in the third only in 1/3 tech. repl.). Statistics: one-way ANOVA with Holm-Šídák’s multiple comparisons test, Welch’s ANOVA with Dunnett’s T3 multiple comparisons test, or Kruskal-Wallis test with Dunn’s multiple comparisons test was applied to compare gene expression levels of each *TAS2R* separately after carboplatin or cisplatin treatment *versus* ctrl solvent. Significant differences are indicated by * *p* ≤ 0.05, ** *p* ≤ 0.01, *** *p* ≤ 0.001, **** *p* ≤ 0.0001.

The ensuing step was to investigate whether carboplatin and cisplatin affect *TAS2R* transcription levels and, consequently, to identify the chemoreceptors potentially involved in the observed changes of cellular bitter response. Therefore, the gene expression of human *TAS2R*s was assessed, as detection at the protein level was not possible due to the lack of specific antibodies. As a result, the gene expression levels (N0) of 12 of the 23 *TAS2R*s analyzed in HGT-1 WT cells were altered due to the treatment with 200 µM carboplatin or 50 µM cisplatin (Fig. 2) in comparison to solvent-treated control HGT-1 WT cells.

Considering the clinically relevant concentration ratio of both Pt-based chemotherapeutic drugs^33^, treatment of the cells with either 200 µM carboplatin or 50 µM cisplatin revealed similar results, with relative effect sizes ranging within 21% to 48% change compared to solvent control. In addition, the gene expression of several TAS2Rs for which Na-HED is a known antagonist, namely *TAS2R31*, *43*, and *50* were altered in carboplatin- or cisplatin-treated HGT-1 WT cells compared to solvent-treated control HGT-1 WT cells. Hence, these in vitro results suggest that Pt-based drugs affect the gene expression of *TAS2R*s in HGT-1 WT cells.

Since carboplatin and cisplatin showed differences in their effect on the cellular bitter response, which might result from gene regulation of *TAS2R*s, the different treatments were also compared. Differences were identified in the changes in gene expression levels (see Supplementary Fig. S2 online) for *TAS2R5* (carboplatin − 27% ± 2% *versus *cisplatin − 47% ± 4%; two-tailed t-test, *p* ≤ 0.001) and *TAS2R46* (carboplatin − 20% ± 9% *versus* cisplatin − 48% ± 8%; two-tailed t-test, *p* = 0.0472). Like *TAS2R5*, *TAS2R4* also belongs to the group of highest expressed *TAS2R*s in HGT-1 WT cells and its expression level was changed after exposure to Pt-based drugs (carboplatin − 25% ± 4% *versus* cisplatin − 37% ± 3%; two-tailed t-test, *p* = 0.051). The changes in *TAS2R4* and *TAS2R5* mRNA contents induced by carboplatin and cisplatin render these chemoreceptors compelling candidates for further investigation of their potential functional role in the cellular bitter response.

### TAS2Rs are involved in the bitter response in HGT-1 cells induced by carboplatin and cisplatin

As *TAS2R4* and *TAS2R5* are among the highest expressed and most regulated *TAS2R*s in carboplatin- and cisplatin-exposed HGT-1 WT cells, and the gene expression of *TAS2R43* – known to be antagonized by Na-HED^26^ – was also regulated (Fig. 2), the functional roles of these bitter taste receptors were investigated. For this purpose, the cellular bitter response induced by either 200 µM carboplatin or 50 µM cisplatin was evaluated in HGT-1 *TAS2R4* KO cells^32^, in HGT-1 *TAS2R5* KD cells, as well as in HGT-1 *TAS2R43* KO cells^26^.

**Fig. 3:**
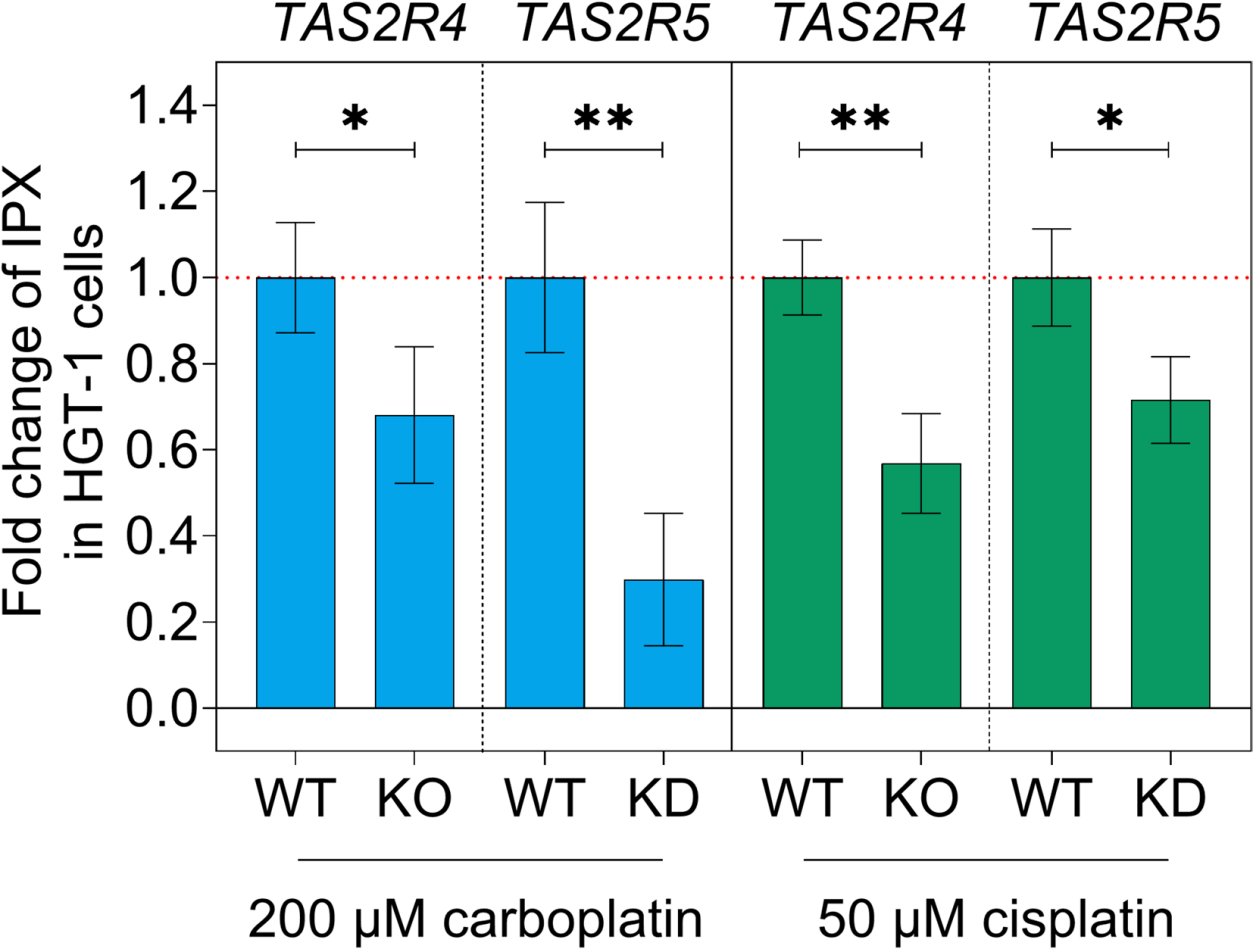
Functional role of TAS2Rs in mediating the cellular bitter response of HGT-1 cells to Pt-based drugs. Fold change of intracellular proton index (IPX) in HGT-1 *TAS2R4* knockout (KO) and HGT-1 *TAS2R5* knockdown (KD) cells after 24-h incubation with 200 µM carboplatin or 50 µM cisplatin compared to the carboplatin- or cisplatin-evoked IPX in HGT-1 wild-type (WT) cells (= 1.0 ± SEM). Data are presented as mean ± SEM, *n* = 3–5, tech. repl. = 4–6; Statistics: one-tailed Mann-Whitney test was used for fold change in carboplatin-treated HGT-1 *TAS2R4* KO cells *versus *HGT-1 WT cells, and one-tailed t-tests for all other comparisons. Significant differences within each treatment group are indicated by * *p* ≤ 0.05, and ** *p* ≤ 0.01.

In comparison to HGT-1 WT cells treated with 200 µM carboplatin (= 1.0 ± 0.13) or 50 µM cisplatin (= 1.0 ± 0.09), the IPX in HGT-1 *TAS2R4* KO cells showed fold changes of 0.68 ± 0.16 (one-tailed Mann-Whitney test, *p* = 0.0257) and 0.57 ± 0.12 (one-tailed t-test, *p* ≤ 0.01), respectively (Fig. 3).

Likewise, the knockdown of *TAS2R5* resulted in a 0.30 ± 0.15-fold carboplatin-induced bitter response (one-tailed t-test, *p* ≤ 0.01) and a 0.72 ± 0.10-fold cisplatin-induced bitter response (one-tailed t-test, *p* = 0.0346) compared to carboplatin- or cisplatin treated HGT-1 WT cells (carboplatin: 1.0 ± 0.17; cisplatin: 1.0 ± 0.11) (Fig. 3). In contrast, the effects on IPX induced by carboplatin or cisplatin in HGT-1 *TAS2R43* KO cells did not differ from those in HGT-1 WT cells (see Supplementary Fig. S1c online), TAS2R43 is unlikely to be involved in this triggered bitter response.

Neither carboplatin nor cisplatin elicited differences in the cellular bitter response between HGT-1 WT and HGT-1 cells treated with transfection reagent only as mock control (see Supplementary Fig. S1b online), thereby excluding possible effects from the knockdown procedure.

Although the CRISPR-Cas9 knockout approach achieved complete deletion of *TAS2R4*^32^, while the knockdown efficiency of *TAS2R5* was only 21.9% ± 8.1% (two-tailed Mann-Whitney test; *p* = 0.024), both approaches resulted in similar fold changes in IPX induced by either 200 µM carboplatin (*TAS2R4* KO: 0.68 ± 0.16 *versus TAS2R5* KD: 0.30 ± 0.15; two-tailed Mann-Whitney test, *p* > 0.05) or 50 µM cisplatin (*TAS2R4* KO: 0.57 ± 0.12 *versus TAS2R5* KD: 0.72 ± 0.10; two-tailed t-test, *p* > 0.05). Hence, TAS2R5 might contribute more substantially to the cellular bitter response induced by Pt-based agents.

### TAS2Rs as well as the presence of the sodium salt of HED modulate cellular uptake of carboplatin and/or cisplatin

Given that (i) exposure to carboplatin and cisplatin elicited a TAS2R-mediated cellular response in our study, and (ii) TAS2R43 was recently shown to co-regulate the ZnCl_2_-derived uptake of the metal ion zinc^28^, we hypothesized that TAS2Rs might also modulate the cellular uptake of Pt-based agents. Alongside the cellular uptake in HGT-1 WT cells, we examined uptake in HGT-1 *TAS2R4* KO and HGT-1 *TAS2R43* KO cells, as the gene expression of both chemoreceptors was regulated by exposure to carboplatin and cisplatin (Fig. 2), and TAS2R4 was additionally functionally involved in sensing these drugs, leading to a cellular bitter response (Fig. 3).

Initially, inductively coupled plasma - mass spectrometry (ICP-MS) analyses were performed to quantitate the cellular uptake of Pt-based drugs in HGT-1 WT cells after 24-h incubation. Carboplatin (50–750 µM) and cisplatin (5–50 µM) were accumulated in HGT-1 WT cells in a concentration-dependent manner, albeit to varying degrees (see Supplementary Fig. S3a online). Despite the difference in concentration between the two Pt-based agents, the comparisons of the cellular uptake after exposing HGT-1 WT cells to either 200 µM carboplatin (50.4 fg Pt/cell ± 1.8 fg Pt/cell) or 50 µM cisplatin (47.3 fg Pt/cell ± 2.0 fg Pt/cell) revealed a similar cellular Pt concentration (see Supplementary Fig. S3a online).

**Fig. 4:**
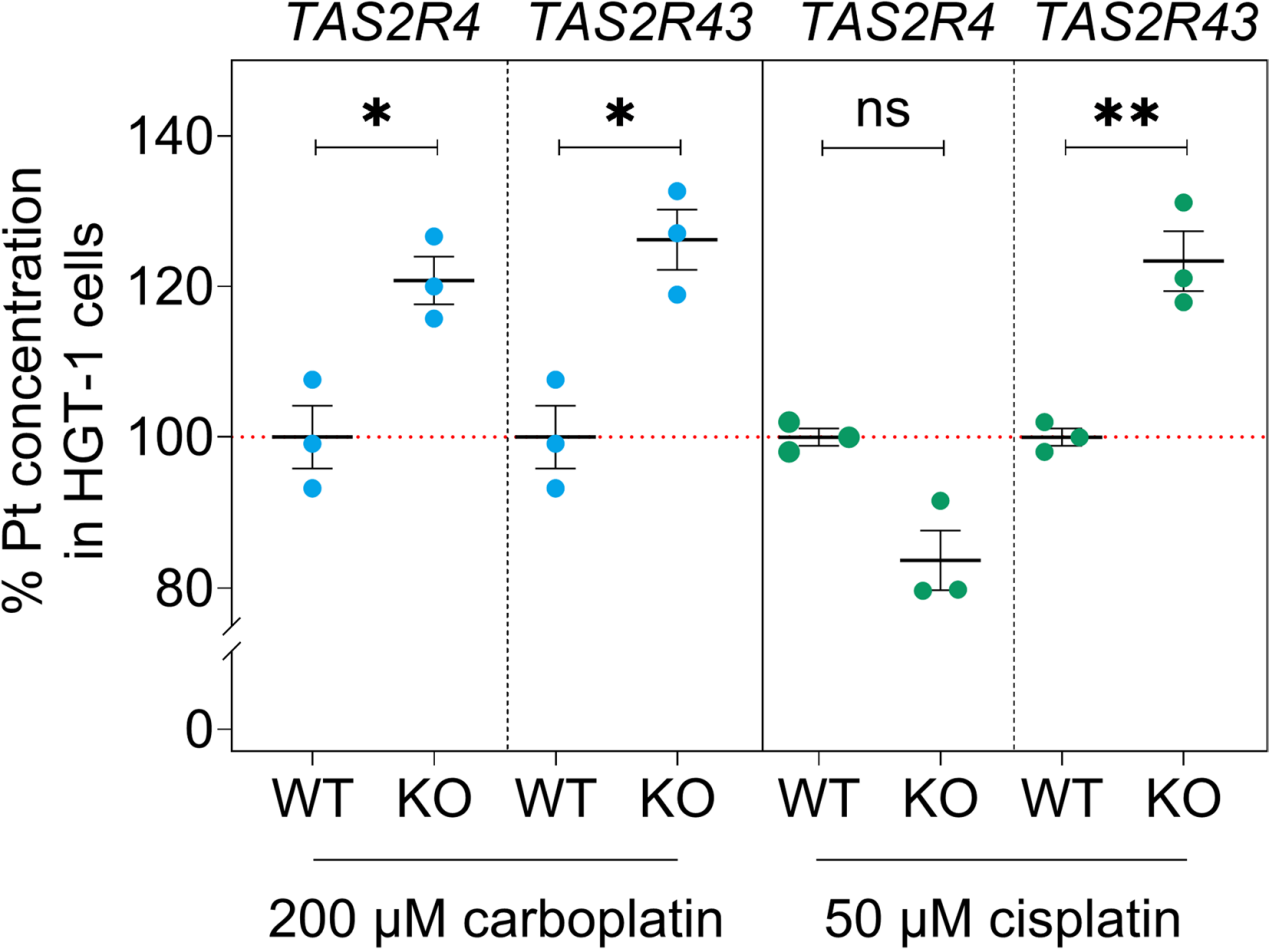
Cellular uptake of carboplatin and cisplatin. Relative intracellular Pt concentration after incubation of HGT-1 *TAS2R4* knockout (KO) and *TAS2R43* KO cells with 200 µM carboplatin or 50 µM cisplatin for 24 h, compared to the cellular Pt concentration in carboplatin- or cisplatin-treated HGT-1 wild-type (WT) cells (= 100% ± SEM). Data are presented as measured values including mean ± SEM, *n* = 3. Statistics: two-tailed Mann-Whitney test was used for relative Pt concentration in cisplatin-treated HGT-1 *TAS2R4* KO cells *versus *HGT-1 WT cells, and two-tailed t-tests for all other comparisons. Significant differences within each treatment group are indicated by * *p* ≤ 0.05 and ** *p* ≤ 0.01, while a non-significant difference (*p* > 0.05) is shown by ns.

Regarding the influence of specific TAS2Rs on the cellular uptake, quantitative analyses of the cellular accumulation of Pt (Fig. 4) showed an increased Pt concentration in HGT-1 *TAS2R4* KO cells after incubation with 200 µM carboplatin (120.8% ± 3.2%; two-tailed t-test, *p* = 0.0166) compared to HGT-1 WT cells (100% ± 4.2%). However, cellular Pt concentration in HGT-1 *TAS2R4* KO cells (83.7% ± 4.0%; two-tailed Mann-Whitney test, *p* = 0.100) was not significantly different from that in HGT-1 WT cells (100% ± 1.2%) after incubation with 50 µM cisplatin.

In HGT-1 *TAS2R43* KO cells, Pt levels were found to be increased after 24-h treatment with 200 µM carboplatin (+ 26.2% ± 4.0%; two-tailed t-test, *p* = 0.0105) and 50 µM cisplatin (+ 23.4% ± 4.0%; two-tailed t-test, *p* ≤ 0.01) *versus* carboplatin- or cisplatin-treated HGT-1 WT cells. Consequently, the absence of TAS2R4 led to increased cellular Pt accumulation after incubation with carboplatin, while TAS2R43 knockout resulted in an augmented uptake of carboplatin and cisplatin, despite the cellular bitter response to both Pt-based agents being mediated by TAS2Rs other than TAS2R43.

In addition, co-incubation of HGT-1 WT cells with 200 µM Na-HED and 200 µM carboplatin or 50 µM cisplatin did not affect the cellular Pt concentration in carboplatin-treated cells, whereas 200 µM Na-HED reduced the cellular uptake of cisplatin by about 23.5% ± 4.4% (two-tailed t-test, *p* ≤ 0.01) (see Supplementary Fig. S3b online). Since the ratios to Na-HED were different between carboplatin (1 + 1) and cisplatin (4 + 1), Nuclear Magnetic Resonance Spectroscopy (NMR) analyses were performed at both molar ratios for both Pt-based drugs to investigate possible molecular interactions with Na-HED. An influence on the chemical shifts of the signals of Na-HED towards higher frequencies (deshielding effect) were only observed for the two mixtures of Na-HED and cisplatin, but not for the mixtures of Na-HED and carboplatin (no interactions) (see Supplementary Table S2 and Fig. S4a-c online). Notably, an equimolar ratio (1 + 1) of Na-HED + cisplatin resulted in a greater shift than a ratio of 4 + 1. Hence, Na-HED does interact with cisplatin and could at least partially counteract the cellular uptake of cisplatin by molecular interactions.

## Discussion

The hypothesis investigated in this study was that Pt-based chemotherapeutic agents activate TAS2Rs, resulting in a cellular bitter response, which is the underlying mechanism for increased sensitivity to bitter taste and bitter phantogeusia in cancer patients receiving Pt-based chemotherapy. In addition to the proposed mechanism, we hypothesized that the bitter-masking flavanone Na-HED reduces these effects on the cellular bitter response and that chemosensory recognition of Pt-based agents by TAS2Rs modulates their cellular uptake.

The primary rationale for focusing on the role of TAS2Rs in chemotherapy-induced bitter taste hypersensitivity and bitter phantogeusia is that clarifying the underlying mechanism is essential for the future development of effective treatment strategies to help affected cancer patients.

To elucidate theses mechanisms, we investigated the effects of carboplatin and cisplatin on the cellular bitter response in HGT-1 cells, an in vitro model for bitterness assessment as a surrogate for sensory analysis^26,30–32^. The in vitro results presented provide evidence that carboplatin and cisplatin target TAS2Rs, leading to a cellular bitter response in HGT-1 WT cells in a dose-dependent manner, but to different extents. Notably, cisplatin requires only one quarter of the carboplatin dose for comparable clinical effectiveness^33^. Accounting for this concentration ratio (1:4), a higher potential of cisplatin to modulate cellular bitter response *via* TAS2Rs was confirmed, implying that a cisplatin-containing chemotherapy may pose a higher risk for increased sensitivity to bitter taste and bitter phantogeusia than a carboplatin-containing treatment.

Although it is known that cisplatin generally has more serious side effects than carboplatin^34^, data about differences in prevalence or magnitude of specific taste dysfunctions between cisplatin- and carboplatin-treated cancer patients are limited and inconsistent^3,35,36^. Apart from a possible agent-specific impact^3,35,37^, the susceptibility to chemotherapy-induced taste disorders can be influenced by several other aspects such as sex^13,37,38^, age^35,37,38^, or even the chemotherapy schedule^36,39^, number of cycles^40^, and number of metastatic sites^39^. Moreover, the use of additional pharmacological agents may also exert an influence, as various drugs are known to cause taste disturbances^41^, and are predicted to^42^ or proven to have a bitter taste^43,44^. Antiemetics, for example, have already been identified as an additional determinant of taste changes in cancer patients^39^, and it is also possible that the combination with other cancer therapeutics has an impact, although this aspect has not yet been sufficiently investigated.

To provide an experimental basis for the future development of effective treatment strategies for increased bitter taste sensitivity and bitter phantogeusia, we tested the impact of the bitter-masking flavanone Na-HED^25^ on the bitter response in HGT-1 WT cells. Na-HED counteracted this response elicited by Pt-based drugs in a dose-dependent manner. Hence, these results provide further evidence for the functional role of TAS2Rs in the bitter reaction triggered by Pt-based chemotherapeutic drugs and highlight Na-HED’s potential as bitter-masking agent to combat bitter phantogeusia in Pt-based chemotherapies.

Both Pt-based compounds regulated the gene expression of several *TAS2R*s, indicating that their effects extend broadly rather than being limited to a single TAS2Rs. Given that 25 human *TAS2R* genes are expressed in taste cells of circumvallate papillae^17^, it seems plausible that Pt-based drugs could exert such a wide-spread effect on TAS2Rs in oral taste cells as well. Supporting our findings that Pt-based drugs regulate gene expression of *TAS2R*s, Tsutsumi et al.^22^ demonstrated that the gene expression level of *TAS2R*5 was elevated in human lingual mucosa of patients experiencing phantogeusia after receiving combined radiochemotherapy with cisplatin and 5-fluorouracil^22^. Additionally, compared to healthy controls, increased expression levels of *TAS2R3*, *42*, and *43* in the mucosal tissue from foliate papillae were found to be related with bitter phantogeusia in non-oncological patients^45^.

The carboplatin- and cisplatin-induced changes we observed in the expression of several *TAS2Rs* prompted us to hypothesize their functional involvement in the bitter response of HGT-1 cells, a hypothesis we subsequently tested using knockout and knockdown experiments. These experiments confirmed that TAS2R4 and TAS2R5, but not TAS2R43, play functional roles in the carboplatin- and cisplatin-elicited bitter response of HGT-1 cells. Although *TAS2R4* was completely knocked out, whereas *TAS2R5* was only partially knocked down, the effect sizes on the cellular bitter response were similar. At this stage, we can only hypothesize possible explanations. One possibility is that the cellular bitter response mediated by TAS2R5 is inherently stronger than that mediated by TAS2R4. Alternatively, we cannot exclude the possibility that other TAS2Rs become more functionally important for chemosensation of an agonist that targets multiple TAS2Rs when one chemoreceptor is completely absent. This may also apply to TAS2R43, whose specific role in sensing Pt-based drugs is not considered essential. Although Na-HED antagonizes TAS2R43^26^ and masks the bitterness of carboplatin and cisplatin in vitro, its antagonistic profile also includes TAS2R20, TAS2R31, and TAS2R50^26^, suggesting that its bitter-masking effect for Pt-based drugs may results from the combined inhibition of multiple TAS2Rs.

Since recent findings by Orth et al.^28,29^ show that gastric TAS2Rs differentially co-regulate the cellular uptake of the metal ions of zinc^28^ and cadmium in HGT-1 cells^29^, we hypothesized that TAS2Rs act as chemosensory regulators of cellular uptake of Pt-based agents as well. To verify this hypothesis, we first showed a compound- and dose-dependent increase of the intracellular Pt concentration in HGT-1 WT cells. Notably, a dose-dependent augmentation of intracellular Pt concentration^46,47^ as well as a higher accumulation in cisplatin-treated compared to carboplatin-treated cells were reported for other cell lines before^46,48^. To our knowledge, our study is the first in vitro study directly comparing cellular Pt concentrations, considering the equivalent concentration ratio between cisplatin and carboplatin (1:4) as applied in clinical practice^33^.

In line with a recent discovery on the co-regulation of cellular zinc uptake^28^, we detected predominantly increased cellular Pt concentrations in HGT-1 *TAS2R4* KO and *TAS2R43* KO cells, suggesting that functional expression of these chemoreceptors reduces the cellular uptake and/or accumulation of cytotoxic Pt-based chemotherapeutic drugs. However, co-incubation with Na-HED – targeting TAS2R20, TAS2R31, TAS2R43, and TAS2R50 as an antagonist^26^, and TAS2R14^26,27^ and TAS2R39 as an agonist^27^ – reduced the cellular uptake of cisplatin, even though the bitter response reflecting cellular sensing was also attenuated. Taking together, a clear link between chemosensory detection of carboplatin and cisplatin and the cellular uptake thereof is not always evident.

Because Pt-based drugs accumulate in cells *via* both passive diffusion and multiple transporters that mediate influx and efflux^49^, TAS2Rs are hypothesized to modulate their uptake *via* transporters, as observed for metal ions such as zinc^28^ and cadmium^29^. Although the specific uptake mechanisms of Pt-based drugs influenced by TAS2Rs remain to be elucidated, the modulatory role of TAS2Rs discovered in this study is particularly significant for oncology, as TAS2Rs are functionally expressed in extraoral cells^50,51^, including both non-cancerous and cancerous cells^52^.

Our observations further point to a complex pattern of involvement of individual TAS2Rs. A similarly nuanced pattern of chemoreceptor-specific functions was recently demonstrated by Orth et al.^29^, showing that TAS2R8 enhances, whereas TAS2R10 protects against excessive cadmium accumulation, while both chemoreceptors exerted corresponding effects on proton secretion in HGT-1 cells^29^. Other factors, such as altered cell membrane stiffness in the absence of the transmembrane chemoreceptor TAS2R43^28^, may also play an additional role, but this needs to be confirmed for Pt-based drugs in future studies. Nevertheless, the presented results provide evidence that TAS2Rs may be implicated in cellular defense against Pt-based chemotherapeutics. Since ensuring effective cellular uptake of cytotoxic chemotherapeutics in cancer cells is a crucial factor for treatment success, the role of TAS2Rs may represent an interesting avenue for future research.

Another underlying, albeit not pivotal, mechanism for the reduced cellular Pt concentration following co-incubation with Na-HED and cisplatin could be molecular interactions between two compounds, as confirmed by NMR analyses. These measurements provide first evidence that Na-HED counteracts, at least partially, the in vitro cellular uptake of cisplatin through molecular interactions, but not that of carboplatin. This raises a new hypothesis that rinsing the mouth with a Na-HED-solution could reduce the uptake and thus the negative effects of cisplatin on taste cell homeostasis and gustatory function which were described by Ren et al.^12^ as well as on salivary glands^53,54^, of which functionality is important for taste perception^55^. Therefore, a localized rinse-and-spit treatment using Na-HED for increased bitter taste sensitivity and bitter phantogeusia could offer multiple benefits: it may specifically alleviate these bitter taste disorders induced by Pt-based chemotherapy and partially reduce the uptake of cytotoxic cisplatin in oral cells, without compromising the efficacy of intravenously administered chemotherapy in cancer cells.

A limitation of the present study is that actual sensory testing of Pt-based drugs would be more ideal to verify bitterness, but given ethical concerns, the in vitro bitterness assay used here remains the best alternative. Although HGT-1 cells express functional TAS2Rs^26^ and their responses to bitter-tasting and bitter-masking compounds can be linked to human bitterness perception^26,30,31^, we acknowledge that an oral taste cell model for bitterness screening – still to be established – could more accurately reflect (i) oral TAS2Rs expression, (ii) activation, and (iii) downstream signaling cascade resulting into a functional response in the tongue’s taste bud cells. In addition to analyzing gene expression alone, it would also be desirable to investigate the protein expression level of TAS2Rs in HGT-1 cells to detect the changes caused by Pt-based agents and sufficient downregulation at the protein level in the knockdown approach. However, specific antibodies still need to be developed for this purpose, which are currently largely lacking in the field of bitter taste receptor research. As TAS2Rs do not interact exclusively with one ligand, but rather with a number of different ligands^18–20^, a further limitation of the study is the lack of highly specific agonists and antagonists for a single TAS2R of interest. Consequently, it is not feasible to achieve classic dose-dependent regular blocking or activation of a specific TAS2R in native cells like HGT-1 cells, which inherently express a range of TAS2Rs^26^.

For future research in this area, we recommend the development of specific antibodies and the investigation of a broad spectrum of chemically diverse chemotherapeutic agents and other drugs commonly used in cancer therapy for potential interactions with TAS2Rs and, in this context, their significance for cellular uptake. Developing a functional oral taste cell model for bitterness assessment could provide further benefits for research on bitter taste disorders, as highlighted earlier in the study’s limitations.

Overall, our in vitro studies demonstrated that TAS2Rs play a functional role in the cellular bitter taste response induced by Pt-based agents, and that Na-HED exhibits a bitter-masking ability against these agents, revealing a novel underlying mechanism and potential treatment option for bitter taste disorders caused by Pt-based chemotherapeutics. These presented in vitro results must be validated in vivo and clinically before an effective treatment option, hypothesized as a bitter-masking mouth rinse containing Na-HED, can be proposed to cancer patients suffering from chemotherapy-induced bitter taste hypersensitivity and bitter phantogeusia.

## Methods

### Chemicals and reagents

Cisplatin (cis-Diammineplatinum (II) dichloride, purity 99.9%), carboplatin (cis-Diammine (1,1-cyclobutanedicarboxylato) platinum, purity 99.6%), penicillin-streptomycin (Pen/Strep; 10,000 units penicillin and 10 mg streptomycin per mL in 0.9% NaCl), trypsin-EDTA solution (1 x, 0.5 g/L porcine trypsin and 0.2 g/L EDTA) and histamine (purity ≥ 97%) were purchased from Sigma-Aldrich (Merck KGaA, Germany). Gibco™ Dulbecco’s Modified Eagle Medium (1x) supplemented with GlutaMAX™-I, high D-glucose and sodium pyruvate (DMEM), Gibco™ fetal bovine serum (FBS), Invitrogen™ 5-(and-6)-carboxy SNARF™-1, acetoxymethyl ester, acetate (SNARF-1 AM) and Invitrogen™ nigericin (free acid) were purchased from Thermo Fisher (Thermo Fisher Scientific Inc., USA). Lonza™ phosphate buffered saline 1x (PBS) was obtained from Biozym Biotech Trading GmbH, Austria. Unless otherwise stated, all other chemicals were acquired from Carl Roth GmbH + Co. KG, Germany, and cell culture plates and flasks from Sarstedt AG & CO. KG, Germany. Na-HED (80.1% purity), isolated in a single batch according to Ley et al. from flavanone-rich leaf wax extracted from dried Herba Santa (*Eriodictyon californicum* (H. & A.) Torr.)^25^, was kindly provided by Symrise AG, Germany.

### Cell culture

The human gastric tumor cell line (HGT-1 wild-type (WT)) was obtained from C. L. Laboisse (Laboratory of Pathological Anatomy, Nantes, France). HGT-1 cells homozygous for *TAS2R4* KO^32^ or *TAS2R43* KO^26^ were previously established, and the knockout was confirmed by sequencing analyses^26,32^. HGT-1 WT (passage number 59–67), HGT-1 *TAS2R4* KO (passage number 27–36), and HGT-1 *TAS2R43* KO (passage number 28–34) cells were cultured in T175 culture flasks in DMEM supplemented with 10% FBS and 1% Pen/Strep (DMEM growth medium) under standard cell culture conditions at 37 °C, 5% CO_2_ and 95% humidity^26^. All incubations of HGT-1 cells with the test compounds for the experiments done in this study were conducted under these cell culture conditions.

### Cell viability

Cell viability was analyzed by means of the 3-(4,5-dimethylthiazol-2-yl-2,5-diphenyl-2*H-*tetrazolium bromide (MTT; thiazolyl blue) assay^32^. A total number of 5.0 × 10^4^ HGT-1 cells per well were seeded in a 96-well plate in DMEM growth medium. After 20–24 h for settling at 37 °C and 5% CO_2_, HGT-1 cells were incubated with carboplatin (50, 100, 200, 500, 750 µM), cisplatin (5, 10, 25, 50, 75 µM), and Na-HED (200 µM) including combinations of Na-HED with carboplatin (200 µM) or cisplatin (50 µM), or with the solvent control (0.9% NaCl, 1:25 dilution) in DMEM growth medium. After 24-h incubation, followed by a washing step with Krebs-Ringer-HEPES buffer (KRHB) identical to that used before measuring intracellular pH in HGT-1 cells, HGT-1 cells were incubated with 100 µL MTT working solution (i.e. 1:6 dilution of MTT-stock (5 mg/mL in PBS)) with serum-free DMEM for 15 min. The absorbance of the formed formazan product, dissolved in 150 µL dimethyl sulfoxide (DMSO; purity ≥ 99,5%), was measured at 570 nm and at a reference wavelength of 650 nm by using Infinite® M200 PRO (Tecan Trading AG, Switzerland) plate reader. Additionally, cell viability was determined for HGT-1 *TAS2R4* KO and HGT-1 *TAS2R43* KO cells, as well as for knockdown approaches of *TAS2R5* in HGT-1 cells. Cell viabilities were calculated in relation to the control cells, which were only treated with medium (ctrl medium = 100%), and the cell viabilities of all experiments are listed in Supplementary Table S1 online.

### Measurement of the intracellular pH as an indicator for cellular bitter response in HGT-1 cells

The intracellular pH in HGT-1 cells, indicating a TAS2R-dependent proton secretion activity^26^, was determined by using the pH-sensitive fluorescence dye SNARF-1 AM, slightly adapted to approaches previously described^32^. 5.0 × 10^4^ HGT-1 cells per well were seeded in a black 96-well plate (Thermo Fisher Scientific Inc., USA) and after 20–24 h at 37 °C and 5% CO_2_, the DMEM growth medium was replaced by 200 µL DMEM growth medium containing carboplatin (50, 100, 200, 500, 750 µM) or cisplatin (5, 10, 25, 50 µM). Concentrations were chosen to allow robust activation of the pathway of interest under conditions that preserved cell viability. The highest effective cisplatin concentration (50 µM) in the TAS2R-dependent proton secretion activity assay was used as the basis for selecting the corresponding carboplatin concentration (200 µM) using a 1:4 ratio^33^. Furthermore, co-treatments with carboplatin (200 µM) and Na-HED (20, 200 µM) as well as cisplatin (50 µM) and Na-HED (5, 20, 50, 200 µM) were performed, representing 1:1 and 1:10 ratios and matching Na-HED concentrations between compounds to avoid causing additional variability. For solvent control (0 µM) and other controls, HGT-1 cells were treated with DMEM growth medium with or without an equal concentration of 0.9% NaCl-solution (1:25 dilution), respectively. HGT-1 cells were treated with the test compounds, combinations thereof or control solution at 37 °C and 5% CO_2_ for 24 h to simulate prolonged exposure of TAS2Rs to Pt-based drugs, given that the drugs can be detected in saliva during and after intravenous Pt-based chemotherapy for several hours^23,24^, with carboplatin remaining detectable for at least 24 h^23^. After this 24-h incubation at 37 °C and 5% CO_2_, HGT-1 cells were washed per well with prewarmed 100 µL KRHB (10 mM 4-(2-hydroxyethyl)-1-piperazineethane-sulfonic acid (HEPES), 11.7 mM D-glucose, 4.7 mM KCl, 130 mM NaCl, 1.3 mM CaCl_2_, 1.2 mM MgSO_4_ and 1.2 mM KH_2_PO_4_, adjusted to pH 7.4). Afterwards, HGT-1 cells were stained with 100 µL of 3 µM SNARF-1 AM in KRHB at 37 °C and 5% CO_2_ for 35 min, then washed twice with KRHB, followed by analysis of the intracellular pH for 10 min after adding 100 µL KRHB per well. To verify a functioning proton secretion in HGT-1 cells, histamine (1 mM) in KRHB was used as a positive control, with KRHB alone serving as a control (ctrl). A calibration curve between pH-values ranging from 7.0 to 8.0 was performed using pH-adjusted KCl-buffer (20 mM HEPES, 18 mM D-glucose, 110 mM KCl, 20 mM NaCl, 1 mM CaCl_2_ and 1 mM MgSO_4_; pH 7.0, 7.2, 7.4, 7.6, 7.8, 8.0) complemented with nigericin (20 µM) to ensure an equivalent intra- and extracellular pH-value. The fluorescence measurements were performed at emission wavelengths of 580 nm and 640 nm after excitation at 488 nm by using the FlexStation^®^ 3 Multi-Mode Microplate Reader (Molecular Devices GmbH, Germany). Using the linear pH-calibration curve and the 580/640 nm ratio, the intracellular pH-value and corresponding intracellular H^+^-concentration, including the ratio between treated and solvent-treated control HGT-1 cells, were calculated from the final measurement at 10 min. This ratio of the intracellular H^+^-concentration was additionally log_2_ transformed to obtain the intracellular proton index (IPX) whereby a reduction of the IPX indicates an increased proton secretion. For co-incubation with Na-HED, the percentage reduction of the carboplatin- and cisplatin-induced effect on IPX was calculated. Since TAS2Rs play a functional role in proton secretion activity as a cellular bitter reaction to bitter-tasting compounds^26,32^, the IPX was determined in HGT-1 *TAS2R4* KO and *TAS2R43* KO cells, where the entire procedure was identical to HGT-1 WT cells, and in HGT-1 *TAS2R5* KD cells, where only the seeding number was different due to the knockdown approach. In the knockdown approach, a normalization step for non-transfected HGT-1 WT cells, HGT-1 mock cells, HGT-1 *TAS2R5* KD cells was conducted to consider the signal difference caused by the knockdown approach itself. Fold changes of the IPX in HGT-1 *TAS2R4* KO, HGT-1 *TAS2R5* KD, HGT-1 *TAS2R43* KO, and HGT-1 mock cells were calculated relative to the mean value of HGT-1 WT cells, which was normalized to 1.0.

### Gene expression analysis of TAS2Rs in HGT-1 WT cells

For analyzing the mRNA expression levels of human *TAS2R*s^26^ in order to investigate the effects of carboplatin and cisplatin on gene expression levels and to identify relevant *TAS2R*s for subsequent knockdown and knockout approaches, 3.5 × 10^5^ HGT-1 WT cells per well were seeded in 6-well plates 20–24 h prior to the treatment. Then, HGT-1 WT cells were incubated with 4 mL of either carboplatin (200 µM) or cisplatin (50 µM) in fresh DMEM growth medium at 37 °C and 5% CO_2_. Solvent-treated control HGT-1 WT cells were only incubated with an equal concentration of 0.9% NaCl (1:25 dilution) in DMEM growth medium. To detect mRNA level changes that precede protein level and functional alterations in the cellular bitter response, HGT-1 cells were treated with the test compounds for 18 h, allowing identification of transcriptomic changes prior to the functional effects measured at 24 h. After an 18-h treatment, HGT-1 WT cells were washed with 1 mL ice-cold PBS, lysed with 600 µL RNA lysis buffer of the Monarch Total RNA Miniprep Kit (New England BioLabs^®^ GmbH, Germany) and the RNA isolation was immediately proceeded following the manufacturer’s protocol. RNA concentration was measured spectrophotometrically at 260 nm and its purity was verified by the OD 260/280 and OD 260/230 ratios using a nanoQuant plate for the Infinite^®^ M200 PRO plate reader (Tecan Trading AG, Switzerland). 1 µg total RNA was used for cDNA synthesis which was conducted with the LunaScript^®^ RT SuperMix Kit (New England BioLabs GmbH, Germany) according to the manufacturer’s protocol including the cycle steps (primer annealing: 2 min/25°C, cDNA synthesis: 10 min/55°C, heat inactivation:1 min/95°C) carried out in the thermocycler (Bio-Rad Laboratories GmbH, Austria). cDNA was 1:10 diluted with nuclease free water. RT-qPCR was performed with the StepOnePlus^™^ Real Time PCR system (Applied Biosystems, Thermo Fisher Scientific Inc., USA) using Luna^®^ Universal qPCR Master Mix (New England BioLabs GmbH, Germany) with primers at a final concentration of 200 nM and the suggested temperature program from the manufacturer (60 s/95°C for initial denaturation, 45 amplification cycles comprising 15 s/95°C for denaturation and 30 s/60°C for extension, melt curve starting from 60 °C to 95 °C (+ 0.5 °C/min)). Sequences of human primers used – each previously validated by sequence analysis – for *TAS2R*s^26^ and for the housekeeping genes *GAPDH* (glyceraldehyde-3-phosphate dehydrogenase), *PPIA* (peptidylprolyl isomerase A), and *TBP* (TATA box binding protein), previously used for gene expression normalization^56,57^, have been published before. Primer pairs were synthesized either by Sigma-Aldrich (Merck KGaA, Germany) or by VBC-Biotech Service GmbH, Austria. By means of LinRegPCR (version 2020.0) the RT-qPCR data was analyzed to determine N0 values, known as the hypothetical initial quantity of specific mRNA. N0 values were normalized to the geometric mean of the three housekeeping genes, namely *GAPDH*, *PPIA* and *TBP*, and the gene expression level of *TAS2R*s was evaluated in relation to the expression level of the solvent-treated control HGT-1 WT cells.

The RNA lysis to prove the efficacy of the knockdown experiment was slightly adapted for the approach in a 96-well plate. Briefly, cells were washed with 200 µL ice-cold PBS, lysed with 200 µL Monarch RNA/DNA protection buffer (1x) and the merged amount of two wells was stored at -80 °C until further RNA isolation following the manufacturer’s instructions. In this approach, only 600 ng RNA were used for cDNA synthesis, which ensured accurate RT-qPCR measurement.

### Knockdown of *TAS2R5* by using small interfering ribonucleic acid (siRNA)

Knockdown experiments were conducted with specific siRNA and HiPerFect transfection reagent (QIAGEN GmbH, Austria). Following the reverse fast-forward protocol of the manufacturer, siRNA/HiPerFect transfection mix (1.00 µL of the respective siRNA, 0.75 µL HiPerFect transfection reagent, 24.25 µL serum-free DMEM) was pipetted into a 96-well plate prior seeding 2.5 × 10^4^ HGT-1 WT cells in 174 µL DMEM growth medium per well, yielding a final volume of 200 µL. The siRNA sequence for *TAS2R5* (sense sequence, 5’-CUCUUACUUCACCUGGUUU[dT][dT]-3’; antisense sequence, 5’-AAACCAGGUGAAGUAAGAG[dT][dT]-3’) was predicted by Rosetta algorithm of Sigma-Aldrich (Merck KGaA, Germany). The final concentration of the specific siRNA targeting the mRNA of *TAS2R5* was 10 nM. Additionally, validation of each knockdown experiment was done using 5 nM of MAPK1 siRNA (sense sequence, 5’-UGCUGACUCCAAAGCUCUG[dT][dT]-3’, antisense sequence 5’-CAGAGCUUUGGAGUCAGCA[dT][dT]-3’) (QIAGEN GmbH, Austria), as a positive control, 10 nM of Allstars Negative Control siRNA (QIAGEN GmbH, Austria), as well as a mock transfection, without any siRNA added but with an equal concentration of HiPerFect transfection reagent (0.75 µL per well). For non-transfected wild-type controls, 2.5 × 10^4^ HGT-1 WT cells were seeded in 200 µL DMEM growth medium in a 96-well plate. After 48 h at 37 °C and 5% CO_2_, the (transfection) medium was replaced by 200 µL DMEM growth medium and HGT-1 cells were incubated for another 24 h before evaluating the knockdown efficacy by means of RT-qPCR.

Knockdown efficiency of *TAS2R5* was 21.9% ± 8.1% (two-tailed Mann-Whitney test; *p* = 0.024), whereas the positive control *MAPK1* was downregulated by about 47.9% ± 1.8% (two-tailed t-test, *p* ≤ 0.0001) compared to HGT-1 WT control cells (*n* = 3, tech. repl. = 3). Mock transfection (+ 19.6% ± 11.8%, two-tailed Welch’s t-test, *p* > 0.05) as well as treatment with scramble siRNA (+ 4.0% ± 9.4%, two-tailed Mann-Whitney test, *p* > 0.05), revealed no effect on gene expression of *TAS2R5* (*n* = 3, tech. repl. = 3). To analyze the proton secretion activity in HGT-1 *TAS2R5* KD cells, transfection medium was replaced either by 200 µL DMEM growth medium alone or by 200 µL DMEM growth medium containing carboplatin (200 µM), cisplatin (50 µM) or an equal amount of 0.9% NaCl-solution (1:25 dilution). To obtain suitable controls for the subsequent determination of IPX, this approach was performed with the same density of HGT-1 cells pretreated with HiPerFect transfection reagent in DMEM growth medium only (mock transfection) and with non-transfected HGT-1 WT cells.

### Cellular uptake of Pt-based chemotherapeutics in HGT-1 cells

In brief, 3.0 × 10^6^ HGT-1 WT cells were cultivated one day in a Corning^®^ culture dish (55 cm^2^ growth area; Sigma-Aldrich; Merck KGaA, Germany) prior to the 24-h treatment at 37 °C and 5% CO_2_ with 12 mL fresh DMEM growth medium containing either carboplatin (50, 100, 200, 500, 750 µM), cisplatin (5, 10, 25, 50 µM), or an equal amount of 0.9% NaCl-solution (1:25 dilution) as a solvent control. Additionally, co-incubations were performed with Na-HED (200 µM), the concentration eliciting the strongest bitter-masking effect in vitro, in combination with either carboplatin (200 µM) or cisplatin (50 µM), as well as with corresponding solvent control. These concentrations and ratios correspond to those used in the evaluation of cellular bitter response in HGT-1 WT cells.

Afterwards, HGT-1 WT cells were washed once with 6 mL prewarmed DMEM and twice with 6 mL prewarmed PBS (1x) (Sigma-Aldrich; Merck KGaA, Germany). After surface detachment using 3.5 mL nonenzymatic cell dissociation solution (Sigma-Aldrich; Merck KGaA, Germany), the complete HGT-1 cell suspension, along with 4.5 mL PBS (1x) used to rinse the cell culture plate, was transferred into a 15 mL tube and centrifuged at 110 g for 15 min at + 25 °C. The supernatant was aspirated, the cell pellet was resuspended in 1 mL PBS (1x) and the cell suspension was passed through a cell strainer (Flowmi^®^, porosity 40 µM; Sigma-Aldrich; Merck KGaA, Germany). The cell number was quantitated by flow cytometry analysis (MACSQuant^®^ Analyzer 16) and cell viability was estimated in parallel using propidium iodide solution (Miltenyi Biotec B.V. & Co. KG, Germany). The procedure was the same for HGT-1 *TAS2R4* KO and *TAS2R43* KO cells, but the 24-h incubation was performed with only 200 µM carboplatin, 50 µM cisplatin and the solvent control (0.9% NaCl-solution, 1:25 dilution with DMEM growth medium). Neither treatment with carboplatin nor with cisplatin showed different effects on cell viability in comparison to control cells (see Supplementary Table S3 online). The cell suspension, for which the number of HGT-1 cells was determined, was then digested to determine the Pt content, as described in the following chapter.

### Determination of the Pt concentration by means of inductively coupled plasma - mass spectrometry (ICP-MS)

Pt concentration was analyzed from HGT-1 WT, *TAS2R4* KO and *TAS2R43* KO cells for the analysis of cellular uptake by ICP-MS^46,58^ using NexION 5000 Multi-Quadruple ICP-MS in combination with Syngistix™ Software for ICP-MS 3.1 (PerkinElmer Inc., USA). The operating conditions of the PerkinElmer^®^ ICP-MS instrument are described in Supplementary Table S4 online. Rhenium (ARISTAR^®^, rhenium standard solution for ICP-MS, VWR International GmbH, Germany) was used as an internal standard. External calibration was performed in the range of 0.25–50 µg/L Pt (TraceCERT^®^, Pt standard for ICP-MS; Sigma-Aldrich; Merck KGaA, Germany) in 2% HNO_3_ (ROTIPURAN^®^ Supra 69%). The limit of quantification (LOQ) was 0.06 µg/L. Seven hundred µL of the HGT-1 cell suspension was transferred to microwaves vessels already filled with 1 mL HNO_3_ (Supra 69%), and 4.3 mL ultrapure water. Cell digestion was conducted in the Anton Paar Multiwave 5000 according to the following temperature protocol: ramp to 190 °C in 20 min, hold 20 min at 190 °C and cool down for 20 min. Prior the ICP-MS analysis the samples were diluted with ultrapure water to 2% HNO_3_. The concentration in femtogram (fg) Pt per cell was calculated based on the concentration of the digested cell suspension measured by ICP-MS and the number of cells previously counted by flow cytometry. The results for HGT-1 *TAS2R4* KO and HGT-1 *TAS2R43* KO were calculated as a percentage of the mean cellular Pt concentration in HGT-1 WT (= 100%).

### Nuclear magnetic resonance spectroscopy (NMR)

NMR spectroscopic PROTON experiments (zg30) were executed on an Avance III 500 MHz system equipped with a cryo-TCI probe (Bruker BioSpin GmbH, Germany) at 27 °C (300 K). Aliquots (600 µL) of each sample were analyzed in 5 × 178 mm NMR tubes (USC tubes, Bruker Switzerland AG, Switzerland). The samples were solved in deuterated dimethyl sulfoxide (DMSO-d6; Merck KGaA, Germany). Data processing and analysis was done using TopSpin Software (version 4.1.3) (Bruker, BioSpin GmbH, Germany).

### Statistical analysis

Data calculations were carried out using Excel (Microsoft 365, Microsoft Corporation, USA) and statistical analyses were conducted with GraphPad Prism 10 (GraphPad Software, Inc., USA). All in vitro data are shown as mean ± standard error of the mean (SEM). Detailed information about the number of biological (n) and technical replicates (tech. repl.) as well as the statistical test used for the respective experiments are stated in the results section.

Before performing one- or two-tailed t-tests, the data were assessed for normality using the Shapiro-Wilk test and homogeneity of variances using the F test. If both assumptions were met, a standard t-test was applied. In cases of non-normal distributions, the Mann-Whitney test was used, whereas unequal variances were handled with Welch’s t-test. For multiple comparisons, an ordinary one-way ANOVA followed by Holm-Šídák’s test was performed when the residuals passed the Shapiro-Wilk normality test and variances were equal according to Brown-Forsythe test. For non-parametric data, the Kruskal-Wallis test with Dunn’s post hoc test was used, and for datasets with unequal variances, Welch’s ANOVA with Dunnett’s T3 multiple comparisons test was applied. Statistical significance was always defined as at least a p-value of ≤ 0.05.

## Supplementary Information

Below is the link to the electronic supplementary material.

Supplementary Material 1

## Data Availability

The data described in the manuscript will be made available upon request and approval by the corresponding author (veronika.somoza@univie.ac.at).

## References

[1] Spotten, L. E. et al. Subjective and objective taste and smell changes in cancer. *Ann. Oncol.* **28**, 969–984. 10.1093/annonc/mdx018 (2017). 28327968 10.1093/annonc/mdx018

[2] Rehwaldt, M. et al. Self-care strategies to Cope with taste changes after chemotherapy. *Oncol. Nurs. Forum*. **36**, E47–E56. 10.1188/09.onf.e47-e56 (2009). 19273394 10.1188/09.onf.e47-e56 PMC2893729

[3] Amézaga, J. et al. Assessing taste and smell alterations in cancer patients undergoing chemotherapy according to treatment. *Support Care Cancer*. **26**, 4077–4086. 10.1007/s00520-018-4277-z (2018). 29855774 10.1007/s00520-018-4277-z

[4] Jeong An, H. & Kang, S. J. The relationship between taste and smell alterations and quality of life among women with breast cancer receiving chemotherapy. *Oncol. Nurs. Forum*. **50**, 499–508. 10.1188/23.Onf.499-508 (2023). 37677751 10.1188/23.ONF.499-508

[5] Sánchez-Lara, K. et al. Influence of taste disorders on dietary behaviors in cancer patients under chemotherapy. *Nutr. J.* **9**, 15. 10.1186/1475-2891-9-15 (2010). 20334666 10.1186/1475-2891-9-15 PMC2858711

[6] Turcott, J. G. et al. Changes in the detection and recognition thresholds of three basic tastes in lung cancer patients receiving cisplatin and Paclitaxel and its association with nutritional and quality of life parameters. *Nutr. Cancer*. **68**, 241–249. 10.1080/01635581.2016.1144075 (2016). 26943275 10.1080/01635581.2016.1144075

[7] Law, M. L. Cancer cachexia: pathophysiology and association with cancer-related pain. *Front. Pain Res.* **3**, 971295. 10.3389/fpain.2022.971295 (2022). 10.3389/fpain.2022.971295 PMC9441771 36072367

[8] Martin, L. et al. Diagnostic criteria for the classification of cancer-associated weight loss. *J. Clin. Oncol.* **33**, 90–99. 10.1200/JCO.2014.56.1894 (2014). 25422490 10.1200/JCO.2014.56.1894

[9] Montazeri, A. Quality of life data as prognostic indicators of survival in cancer patients: an overview of the literature from 1982 to 2008. *Health Qual. Life Outcomes*. **7**, 102. 10.1186/1477-7525-7-102 (2009). 20030832 10.1186/1477-7525-7-102 PMC2805623

[10] Murtaza, B., Hichami, A., Khan, A. S., Ghiringhelli, F. & Khan, N. A. Alteration in taste perception in cancer: causes and strategies of treatment. *Front. Physiol.* **8**, 134. 10.3389/fphys.2017.00134 (2017). 28337150 10.3389/fphys.2017.00134 PMC5340755

[11] Mukherjee, N., Choudhuri, P., Delay, S., Delay, E. R. & R. J. & Cellular mechanisms of cyclophosphamide-induced taste loss in mice. *PLoS One*. **12**, e0185473. 10.1371/journal.pone.0185473 (2017). 28950008 10.1371/journal.pone.0185473 PMC5614555

[12] Ren, W. et al. Cisplatin attenuates taste cell homeostasis and induces inflammatory activation in the circumvallate papilla. *Theranostics* **13**, 2896–2913. 10.7150/thno.81153 (2023). 37284449 10.7150/thno.81153 PMC10240818

[13] Ijpma, I., Timmermans, E. R., Renken, R. J., Horst, T., Reyners, A. K. & G. J. & L. Metallic taste in cancer patients treated with systemic therapy: A questionnaire-based study. *Nutr. Cancer*. **69**, 140–145. 10.1080/01635581.2017.1250922 (2017). 27925850 10.1080/01635581.2017.1250922

[14] Yang, H. H. L. & Lawless, H. T. Descriptive analysis of divalent salts. *J. Sens. Stud.* **20**, 97–113. 10.1111/j.1745-459X.2005.00005.x (2005). 16614749 10.1111/j.1745-459X.2005.00005.x PMC1435863

[15] Ijpma, I., Renken, R. J., Horst, Reyners, A. K. & G. J. & L. Metallic taste in cancer patients treated with chemotherapy. *Cancer Treat. Rev.* **41**, 179–186. 10.1016/j.ctrv.2014.11.006 (2015). 25499998 10.1016/j.ctrv.2014.11.006

[16] Lang, T., Di Pizio, A., Risso, D., Drayna, D. & Behrens, M. Activation profile of tas2r2, the 26th human bitter taste receptor. *Mol. Nutr. Food Res.* **67**, e2200775. 10.1002/mnfr.202200775 (2023). 36929150 10.1002/mnfr.202200775 PMC10239339

[17] Behrens, M., Foerster, S., Staehler, F., Raguse, J. D. & Meyerhof, W. Gustatory expression pattern of the human tas2r bitter receptor gene family reveals a heterogenous population of bitter responsive taste receptor cells. *J. Neurosci.* **27**, 12630. 10.1523/JNEUROSCI.1168-07.2007 (2007). 18003842 10.1523/JNEUROSCI.1168-07.2007 PMC6673335

[18] Meyerhof, W. et al. The molecular receptive ranges of human tas2r bitter taste receptors. *Chem. Senses*. **35**, 157–170. 10.1093/chemse/bjp092 (2010). 20022913 10.1093/chemse/bjp092

[19] Bayer, S. et al. Chemoinformatics view on bitter taste receptor agonists in food. *J. Agric. Food Chem.* **69**, 13916–13924. 10.1021/acs.jafc.1c05057 (2021). 34762411 10.1021/acs.jafc.1c05057 PMC8630789

[20] Ziaikin, E., David, M., Uspenskaya, S., Niv, M. Y. & Bitterdb 2024 update on bitter ligands and taste receptors. *Nucleic Acids Res.* **53**, D1645–D1650. 10.1093/nar/gkae1044 (2024). 10.1093/nar/gkae1044 PMC11701684 39535052

[21] Comeau, T. B., Epstein, J. B. & Migas, C. Taste and smell dysfunction in patients receiving chemotherapy: A review of current knowledge. *Support Care Cancer*. **9**, 575–580. 10.1007/s005200100279 (2001). 11762967 10.1007/s005200100279

[22] Tsutsumi, R. et al. Effects of chemotherapy on gene expression of lingual taste receptors in patients with head and neck cancer. *Laryngoscope* **126**, E103–E109. 10.1002/lary.25679 (2016). 26422579 10.1002/lary.25679

[23] Van Warmerdam, L. J. et al. Monitoring carboplatin concentrations in saliva: A replacement for plasma ultrafiltrate measurements? *Ther. Drug Monit.* **17**, 465–470. 10.1097/00007691-199510000-00006 (1995). 8585109 10.1097/00007691-199510000-00006

[24] Holding, J. D., Lindup, W. E., Roberts, N. B., Salvatori, P. & Stell, P. M. Measurement of platinum in saliva of patients treated with cisplatin. *Ann. Clin. Biochem.* **36**, 655–659. 10.1177/000456329903600515 (1999). 10505218 10.1177/000456329903600515

[25] Ley, J. P., Krammer, G., Reinders, G., Gatfield, I. L. & Bertram, H. J. Evaluation of bitter masking flavanones from herba Santa (eriodictyon Californicum (h. & a.) torr., hydrophyllaceae). *J. Agric. Food Chem.* **53**, 6061–6066. 10.1021/jf0505170 (2005). 16028996 10.1021/jf0505170

[26] I Liszt, K. et al. Caffeine induces gastric acid secretion via bitter taste signaling in gastric parietal cells. *Proc. Natl. Acad. Sci. USA*. **114**, E6260–E6269. 10.1073/pnas.1703728114 (2017). 28696284 10.1073/pnas.1703728114 PMC5544304

[27] Roland, W. S. et al. Bitter taste receptor activation by flavonoids and isoflavonoids: modeled structural requirements for activation of htas2r14 and htas2r39. *J. Agric. Food Chem.* **61**, 10454–10466. 10.1021/jf403387p (2013). 24117141 10.1021/jf403387p

[28] Orth, H. N. et al. Bitter taste receptor tas2r43 co-regulates mechanisms of gastric acid secretion and zinc homeostasis. *Int. J. Mol. Sci.* **26**, 6017. 10.3390/ijms26136017 (2025). 40649796 10.3390/ijms26136017 PMC12249961

[29] Orth, H. N. et al. Bitter taste receptors tas2r8 and tas2r10 reduce proton secretion and differentially modulate cadmium uptake in immortalized human gastric cells. *Int. J. Mol. Sci.* **26**, 9166 (2025). 41009725 10.3390/ijms26189166 PMC12470311

[30] Liszt, K. I., Hans, J., Ley, J. P., Köck, E. & Somoza, V. Characterization of bitter compounds via modulation of proton secretion in human gastric parietal cells in culture. *J. Agric. Food Chem.* **66**, 2295–2300. 10.1021/acs.jafc.7b01051 (2018). 28525714 10.1021/acs.jafc.7b01051

[31] Sterneder, S. et al. Identification of 4′-demethyl-3,9-dihydroeucomin as a bitter-masking compound from the resin of Daemonorops Draco. *J. Agric. Food Chem.* **72**, 20991–20999. 10.1021/acs.jafc.4c04583 (2024). 39277814 10.1021/acs.jafc.4c04583 PMC11440488

[32] Sterneder, S. et al. Astringent Gallic acid in red wine regulates mechanisms of gastric acid secretion via activation of bitter taste sensing receptor tas2r4. *J. Agric. Food Chem.* **69**, 10550–10561. 10.1021/acs.jafc.1c03061 (2021). 34460245 10.1021/acs.jafc.1c03061

[33] Dasari, S. & Bernard Tchounwou, P. Cisplatin in cancer therapy: molecular mechanisms of action. *Eur. J. Pharmacol.* **740**, 364–378. 10.1016/j.ejphar.2014.07.025 (2014). 25058905 10.1016/j.ejphar.2014.07.025 PMC4146684

[34] Oun, R., Moussa, Y. E. & Wheate, N. J. The side effects of platinum-based chemotherapy drugs: A review for chemists. *Dalton Trans.* **47**, 6645–6653. 10.1039/c8dt00838h (2018). 29632935 10.1039/c8dt00838h

[35] Wickham, R. S. et al. Taste changes experienced by patients receiving chemotherapy. *Oncol. Nurs. Forum*. **26**, 697–706 (1999). 10337648

[36] Campagna, S. et al. Prevalence, severity, and self-reported characteristics of taste alterations in patients receiving chemotherapy. *Oncol. Nurs. Forum*. **45**, 342–353. 10.1188/18.ONF.342-353 (2018). 29683116 10.1188/18.ONF.342-353

[37] Özkan, İ., Taylan, S., Eroğlu, N. & Kolaç, N. The relationship between malnutrition and subjective taste change experienced by patients with cancer receiving outpatient chemotherapy treatment. *Nutr. Cancer*. **74**, 1670–1679. 10.1080/01635581.2021.1957485 (2022). 34328368 10.1080/01635581.2021.1957485

[38] Zabernigg, A. et al. Taste alterations in cancer patients receiving chemotherapy: A neglected side effect? *Oncologist* **15**, 913–920. 10.1634/theoncologist.2009-0333 (2010). 20667968 10.1634/theoncologist.2009-0333 PMC3228016

[39] Nolden, A. et al. Co-occurring Gastrointestinal symptoms are associated with taste changes in oncology patients receiving chemotherapy. *J. Pain Symptom Manag*. **58**, 756–765. 10.1016/j.jpainsymman.2019.07.016 (2019). 10.1016/j.jpainsymman.2019.07.016 PMC6823134 31349034

[40] Malta, C. E. N. et al. Risk factors for dysgeusia during chemotherapy for solid tumors: A retrospective cross-sectional study. *Support Care Cancer*. **30**, 313–325. 10.1007/s00520-021-06219-4 (2022). 34283319 10.1007/s00520-021-06219-4

[41] Rademacher, W. M. H. et al. Oral adverse effects of drugs: taste disorders. *Oral Dis.* **26**, 213–223. 10.1111/odi.13199 (2020). 31532870 10.1111/odi.13199 PMC6988472

[42] Dagan-Wiener, A. et al. Bitter or not? Bitterpredict, a tool for predicting taste from chemical structure. *Sci. Rep.* **7**, 12074. 10.1038/s41598-017-12359-7 (2017). 28935887 10.1038/s41598-017-12359-7 PMC5608695

[43] Beltrán, L. R. et al. Reducing the bitter taste of pharmaceuticals using cell-based identification of bitter-masking compounds. *Pharmaceuticals* **15**, 317. 10.3390/ph15030317 (2022). 35337115 10.3390/ph15030317 PMC8953435

[44] Nguyen, H. et al. Worldwide study of the taste of bitter medicines and their modifiers. *Chem. Senses*. bjaf003. 10.1093/chemse/bjaf003 (2025). 10.1093/chemse/bjaf003 PMC12010088 39902731

[45] Hirai, R., Takao, K., Onoda, K., Kokubun, S. & Ikeda, M. Patients with phantogeusia show increased expression of t2r taste receptor genes in their tongues. *Ann. Otol Rhinol Laryngol*. **121**, 113–118. 10.1177/000348941212100208 (2012). 22397221 10.1177/000348941212100208

[46] Ghezzi, A., Aceto, M., Cassino, C., Gabano, E. & Osella, D. Uptake of antitumor platinum(ii)-complexes by cancer cells, assayed by inductively coupled plasma mass spectrometry (icp-ms). *J. Inorg. Biochem.* **98**, 73–78. 10.1016/j.jinorgbio.2003.08.014 (2004). 14659635 10.1016/j.jinorgbio.2003.08.014

[47] Zheng, L. N. et al. Quantitative analysis of gd@c82(oh)22 and cisplatin uptake in single cells by inductively coupled plasma mass spectrometry. *Anal. Bioanal Chem.* **407**, 2383–2391. 10.1007/s00216-014-8422-3 (2015). 25701412 10.1007/s00216-014-8422-3

[48] Song, I. S. et al. Role of human copper transporter ctr1 in the transport of platinum-based antitumor agents in cisplatin-sensitive and cisplatin-resistant cells. *Mol. Cancer Ther.* **3**, 1543–1549. 10.1158/1535-7163.1543.3.12 (2005). 15634647

[49] Zhou, J. et al. The drug-resistance mechanisms of five platinum-based antitumor agents. *Front. Pharmacol.* **11**, 343. 10.3389/fphar.2020.00343 (2020). 32265714 10.3389/fphar.2020.00343 PMC7100275

[50] Lee, S. J., Depoortere, I. & Hatt, H. Therapeutic potential of ectopic olfactory and taste receptors. *Nat. Rev. Drug Discov*. **18**, 116–138. 10.1038/s41573-018-0002-3 (2019). 30504792 10.1038/s41573-018-0002-3

[51] Sternini, C. & Rozengurt, E. Bitter taste receptors as sensors of gut luminal contents. *Nat. Reviews Gastroenterol. Hepatol.***22**, 39–53. 10.1038/s41575-024-01005-z (2025).10.1038/s41575-024-01005-z39468215

[52] Zehentner, S., Reiner, A. T., Grimm, C. & Somoza, V. The role of bitter taste receptors in cancer: A systematic review. *Cancers* **13**, 5891. 10.3390/cancers13235891 (2021). 34885005 10.3390/cancers13235891 PMC8656863

[53] Yamamoto, T. et al. Protective effects of Egcg on salivary gland cells treated with gamma-radiation or cis-platinum(ii)diammine dichloride. *Anticancer Res.* **24**, 3065–3073 (2004). 15517917

[54] Hey, J. et al. Effect of cisplatin on Parotid gland function in concomitant radiochemotherapy. *Int. J. Radiat. Oncol. Biol. Phys.* **75**, 1475–1480. 10.1016/j.ijrobp.2008.12.071 (2009). 19515505 10.1016/j.ijrobp.2008.12.071

[55] Galaniha, L. T. & Nolden, A. A. The role of saliva in taste dysfunction among cancer patients: mechanisms and potential treatment. *Oral Oncol.* **133**, 106030. 10.1016/j.oraloncology.2022.106030 (2022). 35868097 10.1016/j.oraloncology.2022.106030

[56] Rohm, B. et al. Nonivamide, a capsaicin analog, increases dopamine and serotonin release in sh-sy5y cells via a trpv1-independent pathway. *Mol. Nutr. Food Res.* **57**, 2008–2018. 10.1002/mnfr.201200846 (2013). 23929722 10.1002/mnfr.201200846

[57] Tiroch, J. et al. Bitter sensing tas2r50 mediates the trans-resveratrol-induced anti-inflammatory effect on Interleukin 6 release in hgf-1 cells in culture. *J. Agric. Food Chem.* **69**, 13339–13349. 10.1021/acs.jafc.0c07058 (2021). 33461297 10.1021/acs.jafc.0c07058

[58] Theiner, S. et al. The impact of whole human blood on the kinetic inertness of platinum(iv) prodrugs – an hplc-icp-ms study. *Dalton Trans.* **47**, 5252–5258. 10.1039/C7DT04537A (2018). 29560976 10.1039/c7dt04537a PMC5933005

[59] Philip, Pirkwieser Maik, Behrens Veronika, Somoza Metallic Sensation—Just an Off-Flavor or a Biologically Relevant Sensing Pathway? *Journal of Agricultural and Food Chemistry* **69**(6) 1775–1780 10.1021/acs.jafc.0c06463 (2021). 10.1021/acs.jafc.0c06463 33373224

