# Supplementary material for: Chemosensory response to Pt-based chemotherapeutics via bitter taste receptors in vitro reveals a new mechanism for bitter taste disorders
Source: Sci Rep. 2026 Jan 20;16:2634. doi: 10.1038/s41598-026-35636-w (PMC12824329; doi:10.1038/s41598-026-35636-w)
Supplement: Supplementary file 1 — Supplementary Material 1 [file 41598_2026_35636_MOESM1_ESM.pdf]

Supplementary information for the article

Chemosensory response to Pt-based chemotherapeutics *via* bitter taste receptors in vitro reveals a new mechanism for bitter taste disorders

Sofie Zehentner [1,2], Agnes Mistlberger-Reiner [1], Philip Pirkwieser [3], Noreen Orth [3], Valerie Boger [3], Kristin Kahlenberg [3], Johanna Kreißl [3], Christoph Grimm [4], Jakob Peter Ley [5], Veronika Somoza [1,3,6,*]

[1] Institute of Physiological Chemistry, Faculty of Chemistry, University of Vienna, Vienna, Austria.

[2] Vienna Doctoral School in Chemistry (DoSChem), University of Vienna, Vienna, Austria.

[3] Leibniz Institute for Food Systems Biology at the Technical University of Munich, Freising, Germany.

[4] Department of General Gynecology and Gynecologic Oncology, Comprehensive Cancer Center Vienna, Gynecologic Cancer Unit, Medical University of Vienna, Vienna, Austria.

[5] Symrise AG, Research Biobased Ingredients, Research & Technology, Food & Beverage, Taste, Nutrition & Health, Holzminden, Germany.

[6] Chair of Nutritional Systems Biology, School of Life Science, Technical University of Munich, Freising, Germany.

*Correspondence: veronika.somoza@univie.ac.at

The PDF file includes:

Supplementary figures S1 to S4

Supplementary tables S1 to S4

Figure S1 (a-c)

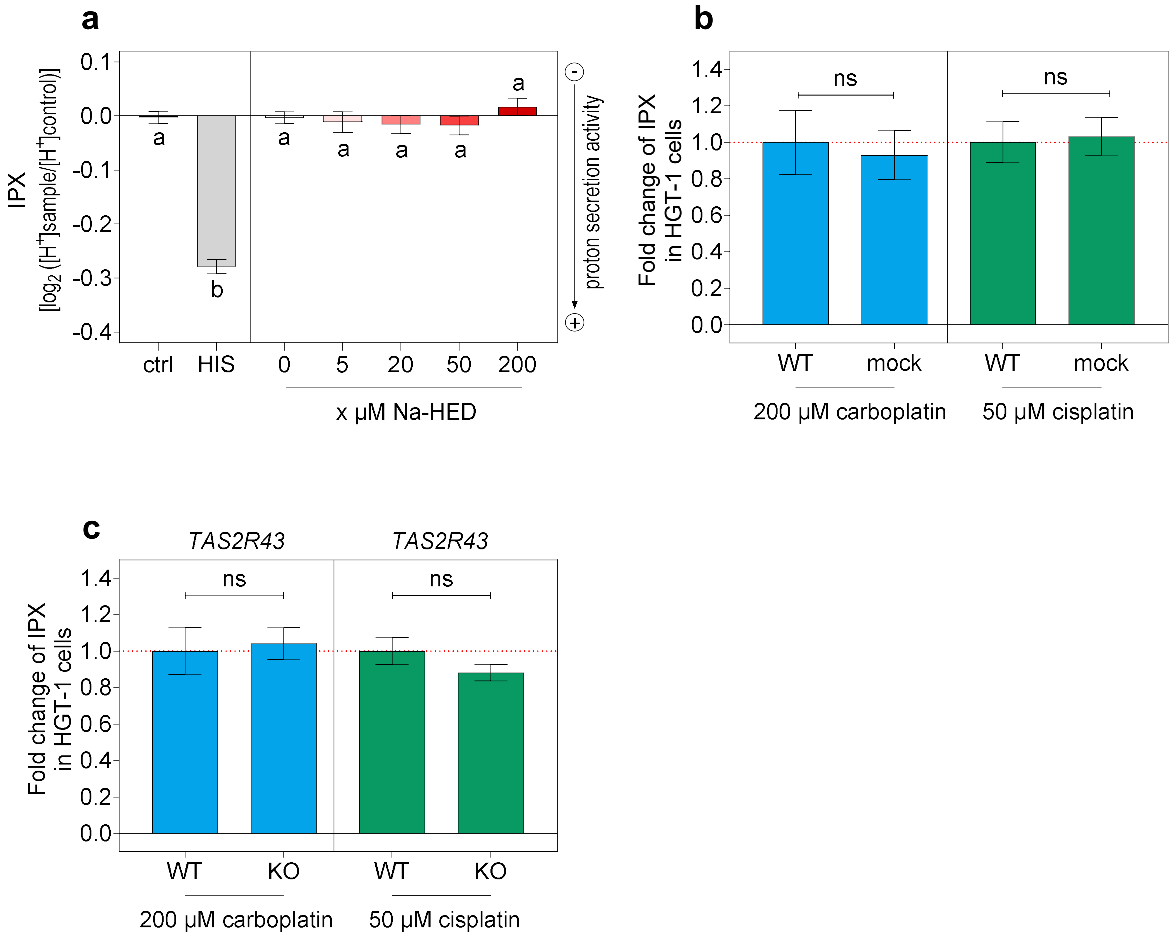

Fig. S1. Proton secretion analyses of HGT-1 cells. **(a)** Intracellular proton index (IPX) of HGT-1 wild-type (WT) cells after incubation with the sodium salt of homoeriodictyol (Na-HED) in various concentrations for 24 h. 1 mM histamine (HIS) was used as positive control for functional H^+-release during measurement, with Krebs-Ringer-HEPES buffer (KRHB) alone serving as a control (ctrl). Data are presented as mean ± SEM, n = 5-15, tech. repl. = 4-6. Statistics: one-way ANOVA with Holm-Šídák's multiple comparisons test for different Na-HED concentrations, and Welch's t-test for ctrl *versus* HIS. Significant differences are indicated by different letters ($p \leq 0.05$). **(b)** Fold change of IPX in HGT-1 mock cells (mock), only treated with HiPerFect transfection reagent, after 24-h incubation with 200 µM carboplatin or 50 µM cisplatin, compared to the carboplatin- or cisplatin-evoked IPX in HGT-1 WT cells (= 1.0 ± SEM). Data are presented as mean ± SEM, n = 3, tech. repl. = 4-6. Statistics: one-tailed t-test for both treatment groups. No significant differences within each treatment group were detected ($p > 0.05$) and are indicated by ns. **(c)** Fold change of IPX in HGT-1 *TAS2R43* knockout (KO) cells after 24-h incubation with 200 µM carboplatin or 50 µM cisplatin, compared to the carboplatin- or cisplatin-evoked IPX in HGT-1 WT cells (= 1.0 ± SEM). Data are presented as mean ± SEM, n = 5, tech. repl. = 9-18. Statistics: one-tailed Welch's t-test for carboplatin treatment, or one-tailed Mann-Whitney test for cisplatin treatment. No significant differences within each treatment group were detected ($p > 0.05$) and are indicated by ns.

Figure S2

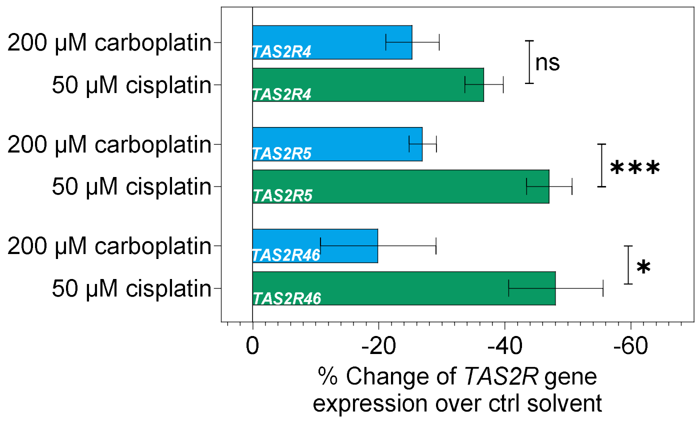

Fig. S2. Differences in changes in gene expression levels for *TAS2R4*, *TAS2R5* and *TAS2R46*. Relative reduction in gene expression levels after 18-h incubation of HGT-1 wild-type (WT) cells with 200 µM carboplatin or 50 µM cisplatin, compared to solvent-treated HGT-1 WT cells (ctrl solvent). Data are presented as mean ± SEM, n = 3, tech. repl. = 2-3. Statistical comparisons were performed between HGT-1 WT cells treated with either 200 µM carboplatin or 50 µM cisplatin, focusing on the percentage reductions in *TAS2R4*, *TAS2R5*, and *TAS2R46* expression relative to solvent-treated cells. Statistics: two-tailed t-tests. Significant differences are indicated by * $p \leq 0.05$, *** $p \leq 0.001$, non-significance is denoted by ns.

Figure S3 (a-b)

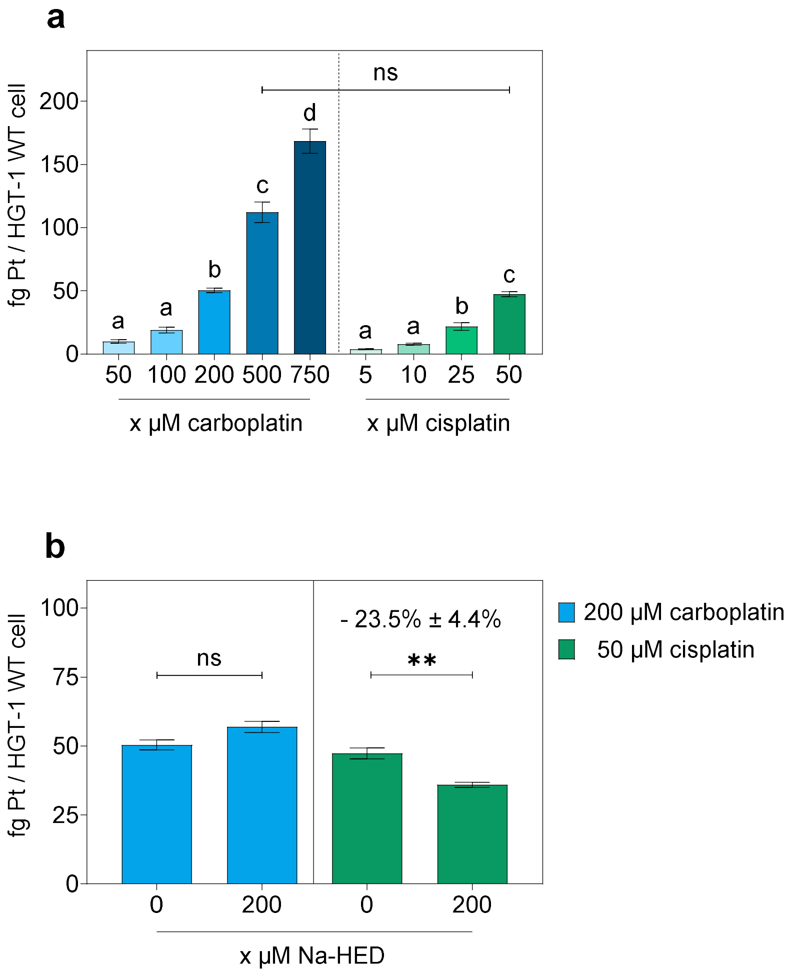

Fig. S3. Cellular uptake of carboplatin and cisplatin. **(a)** Intracellular platinum (Pt) concentration in femtogram (fg) per HGT-1 wild-type (WT) cell measured by means of ICP-MS after incubation of HGT-1 WT cells with carboplatin or cisplatin in various concentrations for 24 h. Data are presented as mean ± SEM, n = 3-4. Statistics: one-way ANOVA with Holm-Šídák's multiple comparisons test, performed separately for the different concentrations of carboplatin and cisplatin, and a two-tailed t-test to compare cellular concentration between cells incubated with 200 µM carboplatin and those incubated with 50 µM cisplatin. Significant differences within each treatment group are indicated by different letters ($p \leq 0.05$), and the non-significant difference between the cellular Pt concentration after incubation with 200 µM carboplatin and 50 µM cisplatin ($p = 0.289$) is indicated by ns. **(b)** Intracellular Pt concentration in fg per HGT-1 WT cell measured by means of ICP-MS after incubation of HGT-1 WT cells with 200 µM carboplatin or 50 µM cisplatin alone and in combination with 200 µM sodium salt of homoeriodictyol (Na-HED) for 24 h. Data are presented as mean ± SEM, n = 4. Statistics: two-tailed t-tests. The p-value for significance was set at ≤ 0.05 and the significant difference is indicated by ** $p \leq 0.01$, while the non-significant difference ($p > 0.05$) is shown by ns.

Figure S4 (a-c)

a

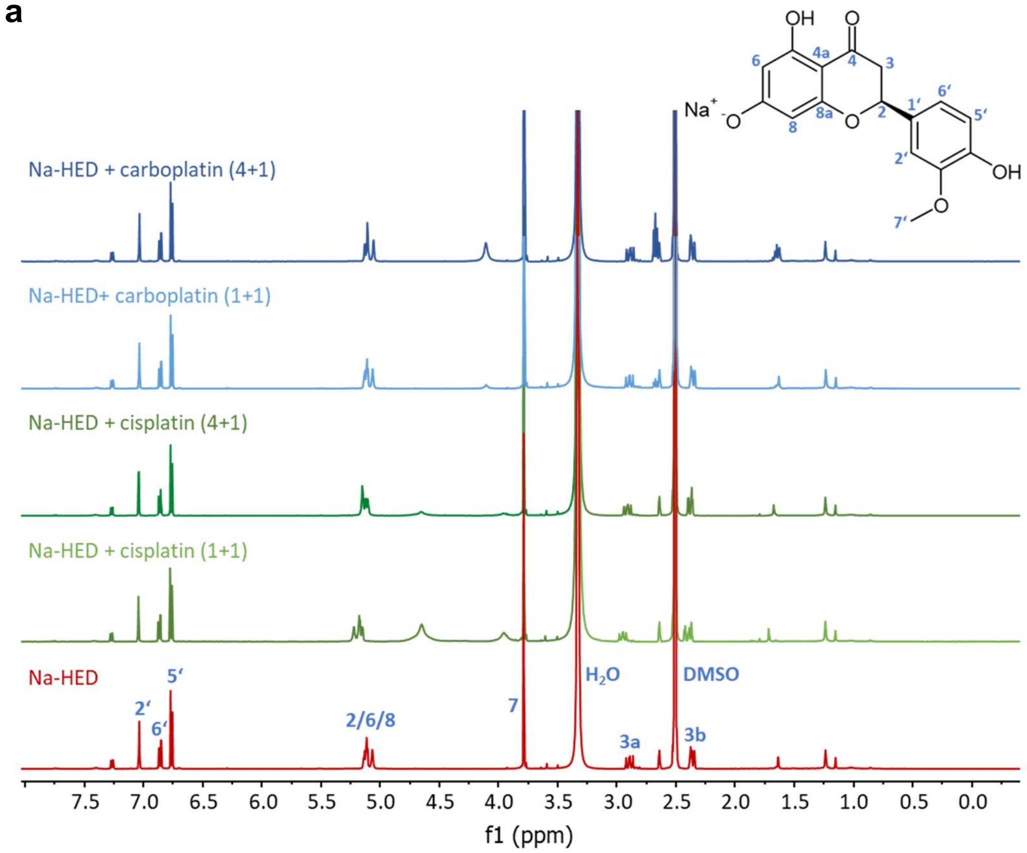

b

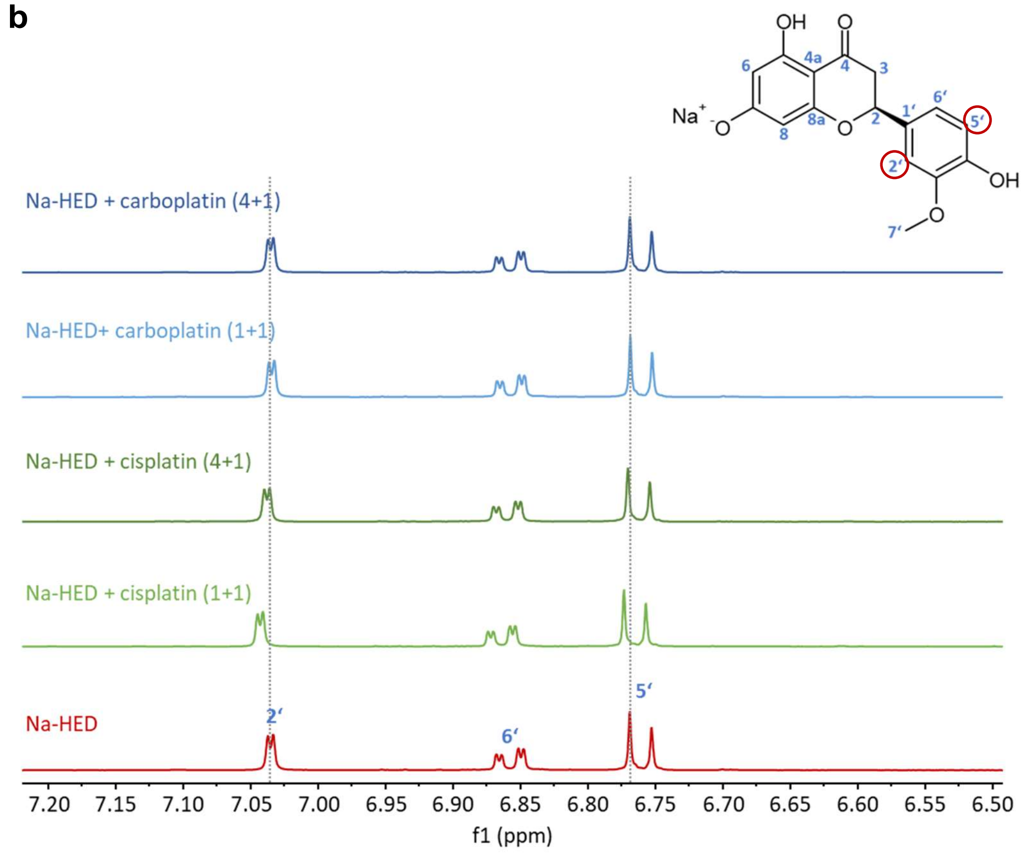

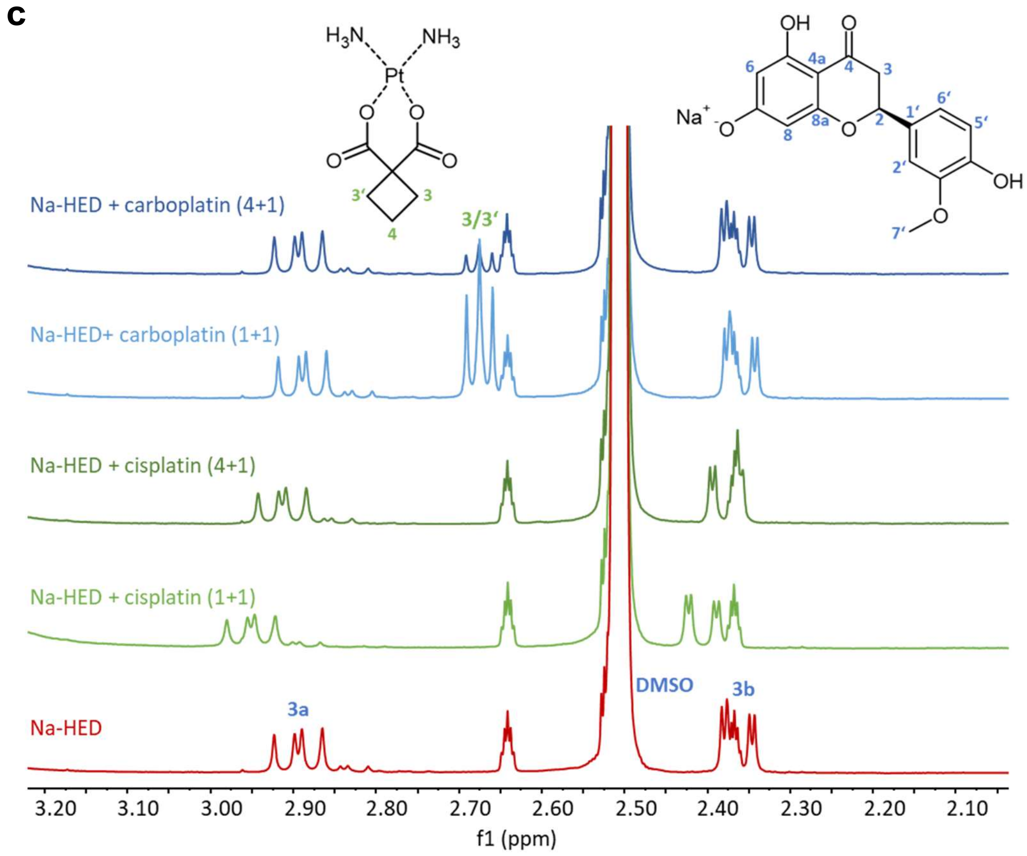

Fig. S4. Molecular interactions between sodium-homoeriodictyol (Na-HED) and cisplatin. **(a-c)** Excerpt of different ^{1}H NMR spectra (500 MHz, zg30, 600 µL sample in DMSO-d_6, 27°C) of Na-HED and mixtures of Na-HED (0.8 mM) and carboplatin or cisplatin in two different molar ratios (4+1 and 1+1).

Table S1. Cell viability of HGT-1 cells determined by means of MTT-assay. Cell viability was calculated as a percentage (%) relative to the medium-treated HGT-1 cells as a control (ctrl medium), which was set to 100%. Data are presented as mean ± SEM, n = 3-12, tech. repl. = 2-4. Statistics: Kruskal-Wallis test with post-hoc Dunn's test or one-way ANOVA with post-hoc Holm-Šídák's test run per experimental group. No significant differences compared to ctrl medium within each group are indicated by ns, while significant differences are indicated by * p ≤ 0.05, ** p ≤ 0.01, **** p ≤ 0.0001.

HGT-1 cells	Treatment	Mean (%)	SEM (%)	
HGT-1 WT cells	ctrl medium	100.0	0.6	
	ctrl solvent	101.5	1.0	ns
	50 µM carboplatin	102.1	1.5	ns
	100 µM carboplatin	102.8	1.7	ns
	200 µM carboplatin	104.8	1.3	ns
	500 µM carboplatin	97.3	1.3	ns
	750 µM carboplatin	91.1	0.6	**
	5 µM cisplatin	99.5	1.2	ns
	10 µM cisplatin	103.8	2.2	ns
	25 µM cisplatin	106.4	1.1	ns
	50 µM cisplatin	104.1	1.2	ns
	75 µM cisplatin	74.8	4.2	***
	200 µM Na-HED	102.4	1.4	ns
	200 µM carboplatin + 200 µM Na-HED	103.6	0.9	ns
	50 µM cisplatin + 200 µM Na-HED	102.4	2.4	ns
HGT-1 *TAS2R4* KO cells	ctrl medium	100.0	1.5	
	ctrl solvent	101.2	1.7	ns
	200 µM carboplatin	94.2	1.2	ns
	50 µM cisplatin	98.0	2.5	ns
HGT-1 *TAS2R43* KO cells	ctrl medium	100.0	0.6	
	ctrl solvent	94.2	1.2	****
	200 µM carboplatin	98.3	0.7	ns
	50 µM cisplatin	80.0	0.9	****
HGT-1 cells (KD approach)	ctrl medium	100.0	3.0	
	siRNA *MAPK1* (5 nM)	91.0	2.3	ns
	negative control (10 nM)	103.6	5.1	ns
	mock (HiPerFect Reagent)	98.9	3.3	ns
	siRNA *TAS2R5* (10 nM)	98.9	3.0	ns
HGT-1 WT cells (ctrl for KD approach)	ctrl medium	100.0	2.0	
	ctrl solvent	103.9	3.2	ns
	200 µM carboplatin	104.3	3.6	ns
	50 µM cisplatin	104.5	1.9	ns
HGT-1 *TAS2R5* KD cells	ctrl medium	100.0	1.8	
	ctrl solvent	107.6	2.3	ns
	200 µM carboplatin	107.9	2.6	ns
	50 µM cisplatin	98.0	2.7	ns
HGT-1 mock cells	ctrl medium	100.0	1.8	
	ctrl solvent	99.3	1.8	ns
	200 µM carboplatin	103.2	2.0	ns
	50 µM cisplatin	100.5	3.5	ns

KD: knockdown; KO: knockout; Na-HED: sodium salt of homoeriodictyol; WT: wild-type

Table S2. Influence on the chemical shifts of sodium salt of homoeriodictyol (Na-HED) and mixtures of Na-HED (0.8 mM) and carboplatin or cisplatin in different molar ratios (4+1 and 1+1).

**Mixture (v:v)**	**Shift difference in *Hz* of the respective proton compared to the signal shift of Na-HED***							
	**H-C2**	**H-C3a**	**H-C3b**	**H-C6/8**	**H-C2'**	**H-C5'**	**H-C6'**	**H-C7'**
Na-HED + carboplatin (4+1)	-1.68	-2.89	-1.57	-4.59	-0.42	-0.34	-0.34	0.00
Na-HED + carboplatin (1+1)	0.00	-0.23	0.00	0.00	0.00	0.00	0.00	0.00
Na-HED + cisplatin (4+1)	6.89	9.74	7.17	18.98	1.39	0.69	1.03	0.00
Na-HED + cisplatin (1+1)	20.66	28.51	21.06	54.25	3.90	2.07	3.22	0.00

*„+“ indicates a more deshielded and „-“ indicates a more shielded proton compared to the signal in the pure solution of HED in DMSO-d6.

Table S3. Cell viability of HGT-1 cells determined by flow cytometry analysis. Cell viability was calculated as a percentage (%) relative to the solvent-treated HGT-1 cells as a control (ctrl solvent), which was set to 100%. Data are presented as mean ± SEM, n = 3-5. Statistics: one-way ANOVA with post-hoc Holm-Šídák's test, run separately for HGT-1 wild-type (WT), TAS2R4 knockout (KO) and TAS2R43 KO cells. No significant differences (ns) compared to ctrl solvent were detected ($p > 0.05$).

**HGT-1 cells**	**Treatment**	**Mean (%)**	**SEM (%)**	
HGT-1 WT cells	ctrl solvent	100.0	1.1	
	50 µM carboplatin	93.4	2.9	ns
	100 µM carboplatin	92.8	2.9	ns
	200 µM carboplatin	95.3	1.7	ns
	500 µM carboplatin	92.9	1.9	ns
	750 µM carboplatin	94.0	3.5	ns
	5 µM cisplatin	95.4	2.4	ns
	10 µM cisplatin	91.5	3.0	ns
	25 µM cisplatin	93.5	1.3	ns
	50 µM cisplatin	92.5	2.8	ns
	200 µM Na-HED	95.4	1.6	ns
	200 µM carboplatin + 200 µM Na-HED	94.2	1.7	ns
	50 µM cisplatin + 200 µM Na-HED	92.9	1.5	ns
HGT-1 *TAS2R4* KO cells	ctrl solvent	100.0	2.0	
	200 µM carboplatin	98.5	0.8	ns
	50 µM cisplatin	100.6	0.8	ns
HGT-1 *TAS2R43* KO cells	ctrl solvent	100.0	0.7	
	200 µM carboplatin	99.2	1.0	ns
	50 µM cisplatin	98.7	0.4	ns

KD: knockdown; KO: knockout; Na-HED: sodium salt of homoeriodictyol; WT: wild-type

Table S4. Operating conditions of the PerkinElmer® ICP-MS instrument.

**Parameter**	**Value**
ICP RF power/W	1600
Nebulizer gas flow (L/min)	0.9
Auxiliary gas flow (L/min)	1.2
Plasma gas flow (L/min)	16
Scan mode (cellular Pt concentration)	MS/MS
IGM	Focusing
Dwell time per AMU (ms)	50
Integration time (ms)	2000
Monitored isotope	^{195}Pt
Monitored isotope of internal standard	^{187}Re

AMU: atomic mass units; ICP RF: inductively coupled plasma radio frequence; IGM: ion guide mode; Pt: platinum; Re: rhenium
